# Virus-Mediated System for Simultaneous Gene Silencing and Genome Editing in Cotton

**DOI:** 10.3390/plants15081153

**Published:** 2026-04-09

**Authors:** Yufeng Zheng, Lianjia Zhao, Yulin Tian, Jiahao Lin, Xiaodong Liu, Jianfeng Lei

**Affiliations:** 1 Innovation Research Center for Efficient Genome Editing Technology and Precision Breeding Applications, College of Life Sciences, Xinjiang Agricultural University, Urumqi 830052, China; 18709006766@139.com (Y.Z.); ts117lin@foxmail.com (Y.T.); 2 Institute of Crop Research, Xinjiang Uyghur Autonomous Region Academy of Agricultural Sciences, Urumqi 830091, China; zlj4537163@163.com; 3 Research Center of Cotton Engineering, College of Agronomy, Xinjiang Agricultural University, Ministry of Education, Urumqi 830052, China; linjiahao0628@163.com; 4 College of Smart Agriculture, Xinjiang University, Urumqi 830046, China

**Keywords:** cotton, TRV, CLCrV, gene silencing, gene editing

## Abstract

Plant viral vectors are powerful tools for the transient expression of exogenous genes, enabling not only virus-induced gene silencing (VIGS) but also virus-induced genome editing (VIGE). However, technical systems capable of simultaneously achieving gene silencing and gene editing in cotton have been rarely reported to date. Therefore, the development of a virus vector system that can concurrently mediate both gene editing and gene silencing would provide a valuable platform for advancing functional genomics studies and molecular design breeding in cotton. To address this gap, we established a system in cotton that concurrently enables gene silencing and gene editing. This system utilizes cotton Cas9 overexpression (Cas9-OE) as a receptor and CLCrV and TRV as vectors for targeting the *GhCLA1* gene, which yields an albino phenotype upon silencing and mutation. Initially, CLCrV and TRV were used independently as vectors for gene editing and gene silencing, respectively. However, our results demonstrated persistent *GhCLA1* gene silencing via TRV, but no systemic gene editing via CLCrV, suggesting viral cross-protection may occur between CLCrV and TRV for simultaneous actions. Subsequently, we constructed tandem assemblies of *GhCLA1* silencing fragments and sgRNA expression elements in both TRV and CLCrV vectors resulted in successful gene silencing and editing, albeit with low editing efficiency. Further optimization through shortening the gene silencing fragments led to a substantial 2.61 to 3.11-fold increase in editing efficiency, while still maintaining effective *GhCLA1* silencing. This refined system provides a robust tool for gene editing in cotton.

## 1. Introduction

Plant viruses co-opt host cell machinery to replicate their genome and to translate viral proteins, making them an ideal tool for delivering exogenous DNA molecules into plants for gene silencing, editing, and overexpression. Viruses such as tobacco rattle virus (TRV) [1,2], cotton leaf crumple virus (CLCrV) [3,4], barley stripe mosaic virus (BSMV) [5,6], and potato virus X (PVX) [7,8] have been employed as vectors for genetic modification, typically delivering ~1–2 kb fragments. However, virus-induced genome editing (VIGE) systems based on these viruses require the use of plants that stably express Cas9 (Cas9-OE) as the receptor. Vectors with large carrying capacities, such as tomato spotted wilt virus (TSWV) [9], sonchus yellow net virus (SYNV) [10] and PVX [11,12], allow for the expression of the complete CRISPR/Cas components required for VIGE without using Cas9-OE as a receptor. The VIGE system not only enables rapid validation of sgRNA efficacy but also induces mutations in target genes in a larger proportion of plant cells as the virus replicates and spreads. This enables efficient functional characterization of genes without necessitating stable genetic transformation.

Despite the advantages of VIGE systems, their application remains limited due to the presence of innate antiviral genes in plants. The expression of these genes inhibits the systemic spread and accumulation of viruses, thereby reducing the efficiency of VIGE in mother plant and potentially eliminating genome editing in gamete cells. For example, genes such as *WUSCHEL* (*WUS*) [13], *PRMT6* [14], and those involved in biosynthesis and signaling pathway of phytohormones, such as abscisic acid [15], salicylic acid [16], gibberellin [17], and jasmonic acid [18], all contribute to plant antiviral defenses. Previously, we demonstrated that CLCrV and TRV viral vectors can mediate targeted editing of endogenous genes in allotetraploid cotton, which comprises A and D subgenomes [4,19]. However, like most plant viruses, CLCrV and TRV-mediated VIGE systems are unable to achieve gene editing in cotton germline cells and achieve heritable editing due to innate antiviral immunity in plant stem cells. Suppression of antiviral genes may enhance viral accumulation and systemic movement within host tissues, thereby improving VIGE efficiency and potentially enabling heritable genome editing. The successful implementation of this strategy depends critically on the establishment of a technical system capable of simultaneously mediating gene silencing and gene editing in cotton.

In addition to their use in VIGE, both DNA and RNA viruses can also be applied for gene silencing [1,3]. This raises the question of whether viral vectors can be engineered to achieve both gene silencing and genome editing simultaneously. Recent studies have shown that CLCrV and TRV can be developed into a multifunctional integrated viral vector toolkit that simplify vector manipulation and enable gene silencing, gene overexpression, and genome editing individually, as well as the concurrent execution of gene silencing and gene overexpression in cotton [20]. However, the aforementioned study did not verify the simultaneous application of VIGS and VIGE in the same plant and the same viral vector system. As mentioned above, there have been few reports of virus vectors that can systemically infect specific plant species to deliver gene silencing fragment and sgRNA expression element—either separately or assembled in tandem within the same vector—to verify the feasibility and efficiency of simultaneously achieving gene silencing and gene editing in a single cotton plant. In this study, CLCrV and TRV were used as viral vectors and Cas9-OE cotton as receptor. *GhCLA1* [1,4,21] was used as a visual reporter gene to enable real-time monitoring of gene silencing efficiency, while *GhCLA1* and the previously identified negative regulator of drought-resistance, *GhAGL16* [22], were selected as target genes for knockout. We successfully developed a viral vector system capable of mediating both gene silencing and genome editing concurrently, providing an effective and practical tool for gene function analysis in cotton.

## 2. Materials and Methods

### 2.1. Plant Materials and Growth Conditions

Wild-type YZ-1 and Cas9-OE cotton seeds [23] with complete germplasm were soaked in double-distilled water (ddH_2_O) for 24 h prior to germination. Seedlings were transplanted into nutrient soil (vermiculite:black soil = 3:1) and grown at 28 °C under a 16 h light/8 h dark photoperiod for approximately 15 d. Seedlings with two fully expanded cotyledons were selected for subsequent inoculation experiments.

### 2.2. Vector Construction

**Figure 1:**
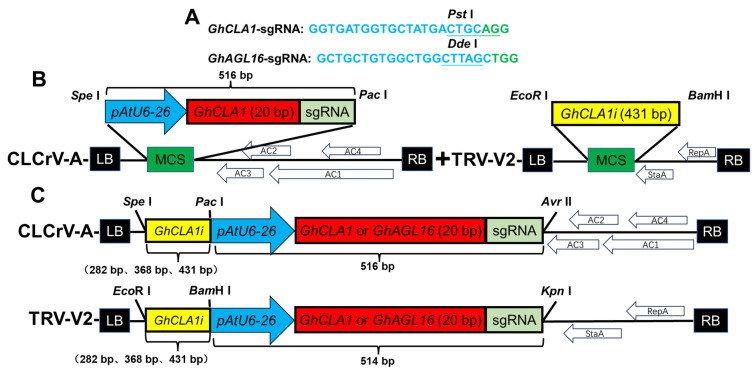
Design of virus-mediated simultaneous gene silencing and gene editing systems in cotton. (**A**) The sgRNAs were designed to target cotton *GhCLA1* and *GhAGL16*, and each target site contained an enzymatic cleavage site to facilitate mutation detection. (**B**) CLCrV-A and TRV-V2 were used to deliver *GhCLA1*-sgRNA and the 431 bp *GhCLA1* gene silencing fragment, respectively. (**C**) Construction of virus vectors for simultaneous *GhCLA1* gene silencing and *GhCLA1* and *GhAGL16* gene editing via CLCrV-A and TRV-V2. AC1: Replication-related protein (Rep); AC2: Transcriptional activator protein (TrAp); AC3: Replication-enhancing protein (Ren); AC4: Unknown functional protein; StaA: Stability-related protein; RepA: Replication-related protein.

*GhCLA1* [1,4], which has a distinct albino phenotype, and the negatively regulated drought-resistance gene *GhAGL16* [22], were selected as targets (Figure 1A). A 431 bp *GhCLA1* gene silencing fragment was cloned from the cDNA of Cas9-OE cotton leaves to serve as a template. Sequencing-validated plasmids harboring AtU6-26::*GhCLA1*-sgRNA [4] and AtU6-26::*GhAGL16*-sgRNA [24] were used as templates to modify the 5′ and 3′ ends the corresponding sgRNA expression vectors, facilitating their recombination with TRV-V2 and CLCrV-A viral vectors. The CLCrV-*GhCLA1*-sgRNA and TRV:*GhCLA1i* vectors were constructed based on CLCrV-A and TRV-V2 virus vectors, respectively (Figure 1B). In addition, the 431 bp *GhCLA1* silencing fragment and sgRNAs that targeted *GhCLA1* and *GhAGL16* were assembled into TRV-V2 and CLCrV-A vectors by restriction ligation to generate the CLCrV:*GhCLA1i*-*GhCLA1*-sgRNA, TRV:*GhCLA1i*-*GhCLA1*-sgRNA, CLCrV:*GhCLA1i*-*GhAGL16*-sgRNA, and TRV:*GhCLA1i*-*GhAGL16*-sgRNA constructs (Figure 1C). The final vectors were transformed into *Agrobacterium* GV3101 competent cells for the inoculation of Cas9-OE plants. All primer designs are shown in Supplementary Table S1.

### 2.3. Virus-Mediated Transient Transformation of Cas9-OE Cotton

**Figure 2:**
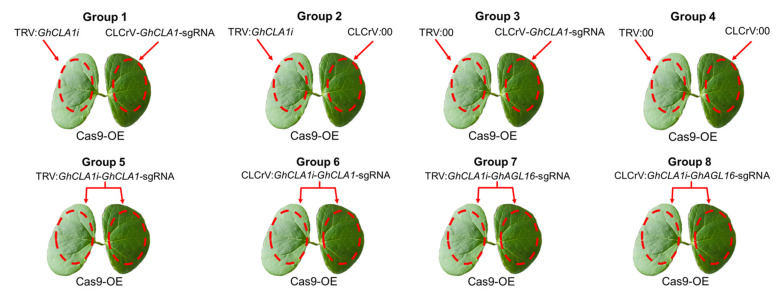
Virus inoculation of Cas9-OE cotton mediated by different viruses.

According to the method of Lei et al. [4], TRV:*GhCLA1i*, TRV-V1, TRV-V2 empty vector, TRV:*GhCLA1i*-*GhCLA1*-sgRNA, TRV:*GhCLA1i*-*GhAGL16*-sgRNA, CLCrV-B, CLCrV-A empty vector, CLCrV-*GhCLA1*-sgRNA, CLCrV:*GhCLA1i*-*GhCLA1*-sgRNA, and CLCrV:*GhCLA1i*-*GhAGL16*-sgRNA were individually scribed for cultivation. The *Agrobacterium* cultures were resuspended in transformation solution (10 mM MgCl_2_, 10 mM MES, 200 μM acetosyringone), adjusted to an OD_600_ of approximately 1.0. For virus inoculation, CLCrV-B was mixed with CLCrV-A empty vector (to form CLCrV:00), CLCrV-*GhCLA1*-sgRNA, CLCrV:*GhCLA1i*-*GhCLA1*-sgRNA and CLCrV:*GhCLA1i*-*GhAGL16*-sgRNA *Agrobacterium* transformant solution in equal proportions. Similarly, TRV-V1 was mixed with TRV-V2 empty vector (to form TRV:00), TRV:*GhCLA1i*, TRV:*GhCLA1i*-*GhCLA1*-sgRNA and TRV:*GhCLA1i*-*GhAGL16*-sgRNA *Agrobacterium* transformant solution in equal proportions. After the above mixtures were left at room temperature for 3 h, the CLCrV-*GhCLA1*-sgRNA mixture and TRV:*GhCLA1i* mixture were inoculated into two cotyledons of the same Cas9-OE plant (Group 1). Using the same inoculation method, we established three control groups (Groups 2–4). In addition, inoculations of two cotyledons of Cas9-OE cotton with TRV:*GhCLA1i*-*GhCLA1*-sgRNA, CLCrV:*GhCLA1i*-*GhCLA1*-sgRNA, TRV:*GhCLA1i*-*GhAGL16*-sgRNA and CLCrV:*GhCLA1i*-*GhAGL16*-sgRNA mixtures were designated Groups 5–8 (Figure 2).

### 2.4. RNA Isolation and qPCR Analysis

RNA was extracted (Transgen, Beijing, China) from the systemic leaves of Cas9-OE plants from each treatment 15–20 days post-inoculation and reverse transcribed into cDNA (Transgen, Beijing, China), which served as template for qPCR amplification (ABI 7500 system, Applied Biosystems, Life Technologies, Carlsbad, CA, USA) of a 143 bp *GhCLA1* fragment, with the internal reference gene *GhUBQ7* as a control. Each sample was analyzed in triplicate. Finally, the relative expression levels of *GhCLA1* were calculated by the 2^−ΔΔCt^ method [25]. In addition, TRV-V2 virus accumulation was detected in systemic leaves of plants from Group 1. All primer designs are shown in Supplementary Table S1.

### 2.5. Mutation Detection

To detect the mutations in *GhCLA1* and *GhAGL16*, genomic DNA was extracted (Transgen, Beijing, China) from the cotyledons and systemic leaves of plants from Groups 1, 3 and 5–8. A 1 µL mixture (62–68 ng/μL) of genomic DNA from all plants from each group was used as a template to detect mutations in *GhCLA1* and *GhAGL16* by PCR/restriction enzyme analysis (PCR/RE) [26]. This process involved PCR amplification of genomic fragments containing the sgRNA binding sites in *GhCLA1* and *GhAGL16*, followed by digestion with the corresponding restriction enzymes at these sites. The undigested PCR products were ligated to the Blunt-Zero cloning vector (Transgen, Beijing, China), and several clones were selected for sequencing to detect mutations in the target sites of *GhCLA1* and *GhAGL16*. In addition, the accumulation of CLCrV-B virus was detected in systemic leaves of inoculated plants from Group 1. All primer designs are shown in Supplementary Table S1.

### 2.6. Gene Editing Efficiency Analysis

To determine the gene editing efficiency of *GhCLA1* and *GhAGL16*, all plants from Groups 5–8 which exhibited *GhCLA1* gene silencing, were subsequently tested for mutations using the PCR/RE method. Meanwhile, high-throughput sequencing primers, tailored to the cloned genome sequences of *GhCLA1* and *GhAGL16* from untreated Cas9-OE plants, were designed (Supplementary Table S1). Hi-TOM high-throughput sequencing [27] was used to determine the real editing efficiency of individual *GhCLA1* and *GhAGL16* mutant lines.

### 2.7. Effects of Different Silencing Fragment Lengths on Gene Silencing and Gene Editing

Silencing fragments of the *GhCLA1* gene, measuring 282 bp and 368 bp, were cloned from Cas9-OE cotton. These fragments were inserted into corresponding viral vectors as follows: TRV:*GhCLA1i*^282bp^-*GhCLA1*-sgRNA, TRV:*GhCLA1i*^368bp^-*GhCLA1*-sgRNA, CLCrV:*GhCLA1i*^282bp^-*GhCLA1*-sgRNA and CLCrV:*GhCLA1i*^368bp^-*GhCLA1*-sgRNA (Groups 9–12). Using the methods described previously, these vectors were inoculated into Cas9-OE plants, and the silencing and editing efficiency of *GhCLA1* in these inoculated plants was determined.

### 2.8. Statistical Analysis

The expression levels of target genes are presented as the mean ± standard deviation (SD) based on three biological replicates. Statistical significance for differences in gene expression was determined using Student’s *t*-test, whereas gene-editing efficiency was analyzed using the Mann–Whitney U test.

## 3. Results

### 3.1. Feasibility Verification of Two Plant Viruses Simultaneously Achieving GhCLA1 Gene Silencing and Gene Editing

**Figure 3:**
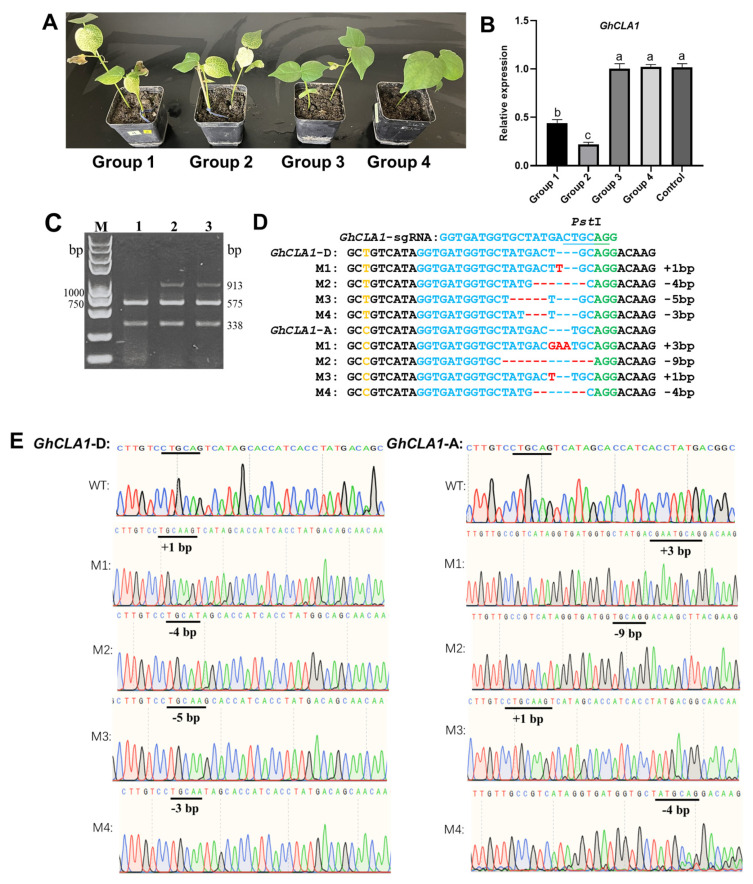
Evaluation of two plant viruses for their ability to simultaneously silence and edit the *GhCLA1* gene in systemic cotton leaves. (**A**) Phenotypic analysis of Cas9-OE cotton plants inoculated with distinct experimental treatments (*n* = 16 plants per group). Fifteen to twenty days post inoculation, systemic leaves of plants inoculated with groups 1 and 2 showed an albino phenotype. (**B**) Detection of *GhCLA1* expression in systemic leaves from Groups 1–4 (*n* = 3). Different lowercase letters indicate significant differences at a 0.05 significance level relative to the control. (**C**) *GhCLA1* mutation detection in infected cotton cotyledons. Lane 1: control; lane 2: Group 1; lane 3: Group 3. (**D**) *GhCLA1* mutation sites in cotton cotyledons from subgenomes A and D. The PAM sequence is highlighted in green, the *Pst* I restriction site within the target sequence is underlined in blue, deletions are marked with short red lines, inserted bases are indicated in red, and bases distinguishing subgenomes A and D are labeled in yellow. (**E**) Sequencing peak map for *GhCLA1* mutations, with M1–M4 indicating different *GhCLA1* mutations.

Previous research demonstrated the feasibility of gene editing in *Nicotiana benthamiana* using two compatible RNA viruses—tobacco etch virus (TEV) and potato virus X (PVX)—to deliver Cas12a and crRNA, respectively [28]. Building on this foundation, we explored a similar system in cotton inoculating distal cotton cotyledons with either a VIGS or a VIGE vector targeting the *GhCLA1* gene, which is known to produce an albino phenotype upon silencing. The experimental groups, outlined in Figure 2, consisted of Groups 1 through 4. Groups 1 (inoculated with CLCrV-*GhCLA1*-sgRNA mixture and TRV:*GhCLA1i* mixture) and 2 (inoculated with TRV:*GhCLA1i* mixture and CLCrV:00 mixture) showed a distinct albino phenotype in systemic leaves after 15~20 d of growth, whereas no systemic albino phenotype was observed in Groups 3 (inoculated with TRV:00 mixture and CLCrV-*GhCLA1*-sgRNA mixture) and 4 (inoculated with TRV:00 mixture and CLCrV:00 mixture) (Figure 3A). qPCR analysis confirmed that *GhCLA1* expression in Groups 1 and 2 was significantly reduced compared to Groups 3 and 4. Furthermore, *GhCLA1* expression levels in Groups 3 and 4 showed no significant difference from those in uninoculated plants (Figure 3B). These results indicate that the downregulation of *GhCLA1* correlates directly with the albinism phenotype.

sgRNA can be systemically expressed in cotton during viral replication and spread, such that cotton cotyledons inoculated with sgRNA expressing vectors serve as the initial site of expression. Cotyledon genomic DNA of 4~5 plants from either Group 1 or 3 was pooled. This mixed DNA, along with DNA from untreated cotton, was used as the template to evaluate *GhCLA1* mutations via PCR/RE [26]. In the cotyledons of Groups 1 and 3, the *GhCLA1* amplification products were not completely digested by *Pst* I, whereas in the control (uninoculated Cas9-OE plant), they were fully digested into two bands (575 bp and 338 bp) without any residual amplification products (Figure 3C). Further cloning and sequencing of undigested *GhCLA1* amplification products from Groups 1 and 3 revealed that *GhCLA1* in cotton subgenomes A and D exhibited various mutation types at the target site, including deletions of 3, 4, 5 and 9 bp and insertions of 1 and 3 bp (Figure 3D,E).

We further analyzed mutations in systemic leaves of Group 1, which exhibited the albino phenotype, to explore potential simultaneous systemic silencing and editing events in the *GhCLA1* gene. *GhCLA1* amplification products from systemic leaves of Groups 1 and 3 were fully digested by *Pst* I (Supplementary Figure S1). In addition, amplification of TRV-V2 and CLCrV-B viral genome sequences in systemic leaves revealed only the accumulation of TRV-V2 virus, with no detection of CLCrV-B virus (Supplementary Figure S2). These results indicate that, within the same cotton plant, TRV-mediated delivery of a *GhCLA1* gene silencing fragment can consistently achieve gene silencing in systemic leaves, whereas CLCrV-mediated delivery of *GhCLA1*-sgRNA failed to edit the *GhCLA1* gene systemically.

### 3.2. Establishment of a Tandem Gene Silencing and Editing System in Cotton

**Figure 4:**
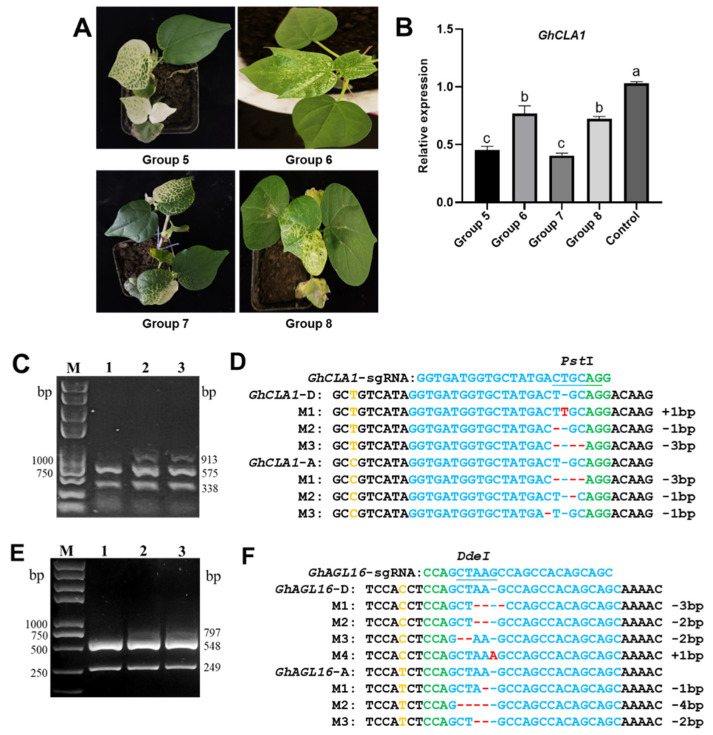
Establishment of a simultaneous gene silencing and editing system in cotton. (**A**) Phenotypic analysis of Cas9-OE cotton plants inoculated with distinct experimental treatments (*n* = 12 plants per group). Fifteen to twenty days post inoculation. Systemic leaves from Groups 5 and 7 showed albino phenotypes, whereas Groups 6 and 8 showed a yellowing phenotype. (**B**) Detection of *GhCLA1* expression in Groups 5–8 (*n* = 3). Different lowercase letters indicate significant differences at the 0.05 level compared with the control. (**C**) Detection of *GhCLA1* mutations. Lane 1: control; lane 2: Group 5; lane 3; Group 6. (**D**) Types of *GhCLA1* mutations in subgenomes A and D. (**E**) Detection of *GhAGL16* mutations in Groups 7 and 8. Lane 1: control; lane 2: Group 7; lane 3: Group 8. (**F**) Types of *GhAGL16* mutations in subgenomes A and D. The PAM sequence is highlighted in green, the restriction site within the target sequence is underlined in blue, deletions are marked with short red lines, inserted bases are indicated in red, and bases distinguishing subgenomes A and D are labeled in yellow.

To further explore the potential for simultaneously achieving gene silencing and gene editing in cotton, we constructed a tandem assembly of a 431 bp *GhCLA1* silencing fragment with sgRNAs targeting either *GhCLA1* or *GhAGL16* into TRV-V2 and CLCrV-A vectors. This setup is detailed in Figure 2, Groups 5–8. Fifteen to twenty days post inoculation, all plants in Groups 5 (inoculated with TRV:*GhCLA1i*-*GhCLA1*-sgRNA mixture) and 7 (inoculated with TRV:*GhCLA1i*-*GhAGL16*-sgRNA mixture) showed an obvious albino phenotype, whereas Groups 6 (inoculated with CLCrV:*GhCLA1i*-*GhCLA1*-sgRNA mixture) and 8 (inoculated with CLCrV:*GhCLA1i*-*GhAGL16*-sgRNA mixture) showed a yellowing phenotype (Figure 4A). *GhCLA1* expression in Groups 5 and 7 was 5-fold lower than that of the control group, and down-regulated by approximately 1.2-fold in Groups 6 and 8 (Figure 4B), consistent with the observed phenotypes.

PCR/RE analysis revealed weak residual amplification products in *GhCLA1* from Groups 5 and 6 after *Pst* I digestion (Figure 4C). Sequencing of these undigested *GhCLA1* amplification products identified various mutation types including base insertions (1 bp) and deletions (1 bp and 3 bp) in subgenomes A and D (Figure 4D and Supplementary Figure S3). In addition, incomplete digestion of *GhAGL16* amplification products by *Dde* I was detected in systemic leaves from Groups 7 and 8 (Figure 4E). Sequencing results showed that *GhAGL16* also exhibited various mutations, including base insertions (1 bp) and deletions (1, 2, 3 and 4 bp) in subgenomes A and D (Figure 4F and Supplementary Figure S4). The above results indicate that tandem assembly of gene silencing fragments and sgRNA expression elements in TRV or CLCrV can achieve simultaneous gene silencing and editing within the same Cas9-OE cotton plant.

### 3.3. Assessment of Gene Editing Efficiency

**Figure 5:**
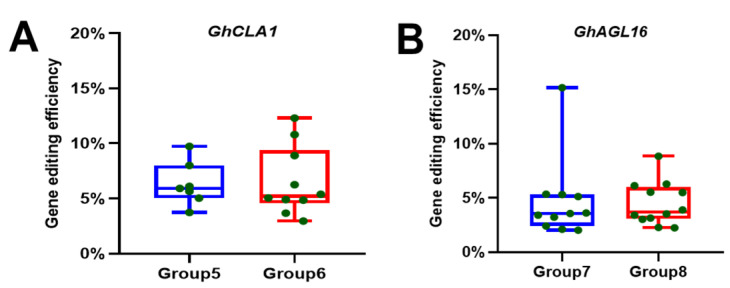
Comparison of efficiency between TRV- and CLCrV-mediated simultaneous gene silencing and editing systems in cotton. (**A**) Statistical analysis of *GhCLA1* gene editing efficiency based on Group 5 and 6. (**B**) Statistical analysis of *GhAGL16* gene editing efficiency based on Group 7 and 8.

To quantitatively assess the efficiency of simultaneous gene silencing and editing for both *GhCLA1* and *GhAGL16* in systemic leaves, mutation detection was conducted on Groups 5–8. PCR/RE analysis showed that mutations in the *GhCLA1* gene occurred in 7 out of 12 plants in Group 5, and 10 out of 11 in Group 6. For the *GhAGL16* gene, all inoculated plants in Groups 7 and 8 carried mutations (Supplementary Figure S5). Hi-TOM high-throughput sequencing was further conducted on these single mutant lines. Deep sequencing analysis showed that each mutant plant contained multiple distinct mutant alleles, indicating that all these individuals were chimeric. The editing efficiencies for *GhCLA1* in Group 5 and Group 6 ranged between 3.60~9.74% and 2.96~12.31%, respectively. The editing efficiencies for *GhAGL16* in Group 7 and Group 8 ranged between 2.02~15.15% and 2.24~8.84%, respectively (Figure 5 and Supplementary Table S2). These results indicate that the tandem system can effectively achieve simultaneous gene silencing and editing when delivered by either CLCrV and TRV, without significant differences in editing efficiency of the same target gene across the delivery method. However, both viruses exhibited relatively low editing efficiencies in systemic leaves.

### 3.4. Effects of Different Silencing Fragment Length on Gene Silencing and Editing Efficiencies

**Figure 6:**
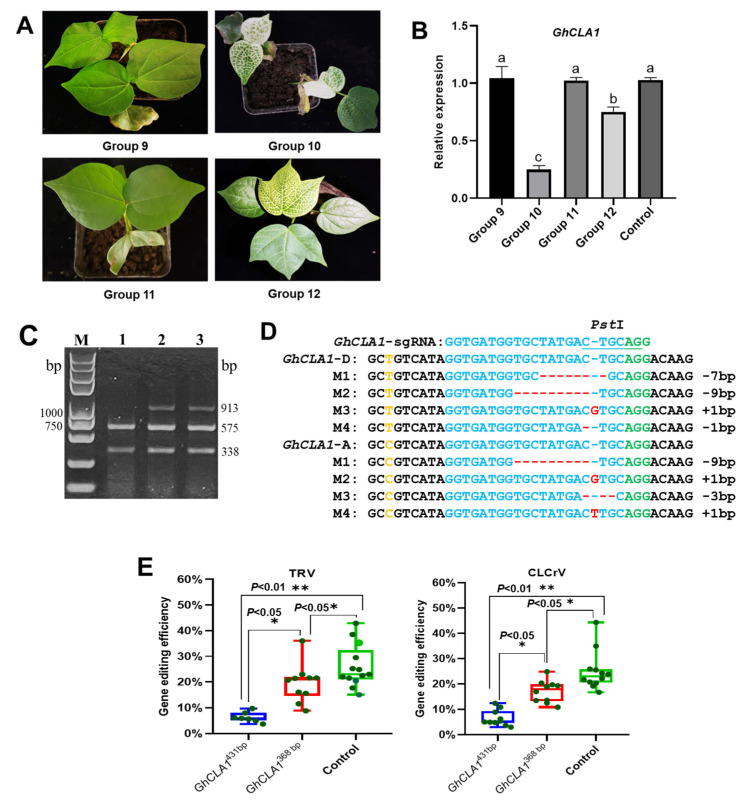
Effects of silencing fragment length on gene silencing and editing efficiencies. (**A**) Inoculation of distinct experimental groups on Cas9-OE cotton plants. Fifteen to twenty days post inoculation, systemic leaves from Group 10 showed albino phenotypes, whereas those from Group 12 showed a yellowing phenotype. (**B**) Expression of *GhCLA1* in Groups 9–12 (*n* = 3). Different lowercase letters indicate significant differences at the 0.05 level compared with the control. (**C**) Detection *GhCLA1* mutations. Lane 1: control; lane 2: Group 12; lane 3: Group 10. (**D**) Types *GhCLA1* mutations in subgenomes A and D. The PAM sequence is highlighted in green, the *Pst* I restriction site within the target sequence is underlined in blue, deletions are marked with short red lines, inserted bases are indicated in red, and bases distinguishing subgenomes A and D are labeled in yellow. (**E**) Statistical analysis of *GhCLA1* gene editing efficiency by different silencing fragment lengths based on TRV and CLCrV virus vectors. * indicate significant differences at the 0.05 level, ** indicate significant differences at the 0.01 level.

When TRV and CLCrV are used as VIGS vectors, gene silencing efficiency improves with increased length of exogenous DNA fragments, leading to a gradual enhancement of the silencing phenotype. However, silencing is completely lost when silencing fragment sizes exceed 1000 bp due to their carrying capacity [3,29]. There is a possibility that the increase in VIGS fragments may result in less efficient gene editing and vice versa. To verify this hypothesis and further improve gene editing efficiency, we cloned two short *GhCLA1* gene silencing fragments of 282 bp and 368 bp, and constructed virus vectors for simultaneous silencing and editing of *GhCLA1* via TRV and CLCrV (Groups 9–12) (Supplementary Figure S6). Fifteen to twenty days after inoculating Cas9-OE plants with these expression vectors, all plants in Group 10 (inoculated with TRV:*GhCLA1i*^368bp^-*GhCLA1*-sgRNA mixture) exhibited obvious albino phenotypes, while some plants in Group 12 (inoculated with CLCrV:*GhCLA1i*^368bp^-*GhCLA1*-sgRNA mixture) developed a yellowing phenotype; no silencing phenotype was observed in Groups 9 (inoculated with TRV:*GhCLA1i*^282bp^-*GhCLA1*-sgRNA mixture) and 11 (inoculated with CLCrV:*GhCLA1i*^282bp^-*GhCLA1*-sgRNA mixture). This observation is consistent with the patterns of *GhCLA1* expression (Figure 6A,B). In addition, two viral vectors lacking sgRNA expression cassette were constructed via CLCrV and TRV, including CLCrV:*GhCLA1i*^282bp^ and TRV:*GhCLA1i*^282bp^. Phenotypic characterization and quantitative analysis of *GhCLA1* transcript abundance revealed that, consistent with the results obtained using vectors carrying both the 282 bp silencing fragment and sgRNA expression cassette, plants inoculated with these two vectors showed neither significant down-regulation of *GhCLA1* expression nor obvious gene silencing phenotypes (Supplementary Figure S7). We found that the 282 bp silencing fragment was ineffective in inducing *GhCLA1* gene silencing mediated by either TRV or CLCrV.

Subsequent experiments detected *GhCLA1* mutations in systemic leaves in Groups 10 and 12, where PCR/RE and sequencing confirmed that both viruses induced mutagenesis (Figure 6C,D and Supplementary Figure S8). Furthermore, we inoculated Cas9-OE plants with TRV-*GhCLA1*-sgRNA and CLCrV-*GhCLA1*-sgRNA viral vectors (controls) without inserting any silencing fragments and tested the editing efficiency of *GhCLA1* in systemic leaves. Hi-TOM high-throughput sequencing showed that *GhCLA1* gene editing efficiencies for Group 10 and Group 12 ranged between 8.87–36.00% and 10.82–24.80%, respectively. In the control group, the editing efficiencies of TRV-*GhCLA1*-sgRNA and CLCrV-*GhCLA1*-sgRNA were 15.00–42.88% and 16.67–44.34%, respectively (Supplementary Table S3). All the mutant plants were confirmed to be chimeras. Compared with Groups 5 and 6, expression of the 368 bp *GhCLA1* silencing fragment led to increases of 3.11-fold (TRV) and 2.61-fold (CLCrV), respectively, in gene editing efficiency (Figure 6E). Compared to the 431 bp silencing fragment, the 368 bp *GhCLA1* silencing fragment not only efficiently silenced *GhCLA1* but also improved the efficiency of *GhCLA1* gene editing mediated by TRV and CLCrV.

## 4. Discussion

CRISPR gene editing technology is a powerful tool for the precise manipulation of plant genomes and the development of germplasms with desirable traits. Achieving heritable gene editing requires the efficient delivery of CRISPR components into germ cells or embryogenic cells, which remains a key objective in current research. However, cotton presents unique challenges for CRISPR application due to limitations associated with transformation and genotype-specific constraints. The VIGE system offers a promising alternative, as certain plant viral vectors are capable of directly or indirectly editing the plant shoot apical meristem (SAM) tissues to facilitate heritable gene modifications. The SAM comprises a population of stem cells that give rise to all aerial tissues and organs, including germline cells. Consequently, successful targeted genome editing within the SAM represents a critical prerequisite for achieving heritable gene modifications through VIGE approaches. For example, BSMV has demonstrated the ability to colonize SAM tissues. When Cas9-OE wheat and barley were inoculated with BSMVγ harboring suitable sgRNAs, heritable gene editing was achieved, although mutation efficiencies were unstable [6,30,31,32]. Additionally, the integration of mobile RNA elements such as *FT* and tRNA^Ileu^ with sgRNA, when assembled in TRV, successfully facilitated the systemic transport of sgRNAs into SAM tissues in *Nicotiana benthamiana*, yielding heritable gene editing with high efficiency [2]. Similarly, fusing tRNA^Ileu^ with sgRNA or enhanced sgRNAs (esgRNAs) in TRV vectors has also proven to be an efficient method for obtaining heritable gene-edited progeny in *Arabidopsis* [33,34]. Editing of target genes in Cas9-OE cotton using CLCrV and TRV-mediated VIGE systems has been successfully demonstrated [4,19,24,35]. However, to date, heritable gene-edited progeny has not been obtained, likely due to the presence of innate antiviral genes in cotton, especially the *WUS* gene. Therefore, a virus vector system capable of achieving both gene editing and gene silencing simultaneously would represent a powerful tool for advancing VIGE applications. However, *GhWUS* is highly and specifically expressed in SAM tissues [36], making its transcript levels difficult to quantify following silencing. For this reason, *GhCLA1* was selected as a visual reporter gene to monitor gene silencing efficiency in this study.

We first explored the feasibility of using two different plant viruses, inoculated into distal cotton cotyledons, to achieve simultaneous silencing and editing of the *GhCLA1* gene in systemic leaves. We found that in systemic leaves of the same Cas9-OE cotton plant, TRV successfully silenced *GhCLA1* whereas CLCrV failed to infect and deliver *GhCLA1*-sgRNA to induce editing events (Figure 3). Previous studies have reported the successful use of two RNA viruses (TEV and PVX) to deliver Cas12a and crRNA to the same cell in *Nicotiana benthamiana*, where they efficiently edited the *NbXT1* and *NbFT* genes [28]. Similarly, the use of tobacco ringspot virus (TRSV) to deliver Cas9 and TRV to deliver sgRNA in *Nicotiana benthamiana* can also play a synergistic role in achieving target gene editing [37]. In this study, the inability of CLCrV and TRV to function synergistically may be attributed to viral cross-protection between the two viruses. During viral systemic spreading, the two viral vectors mixed at an equal ratio compete to infect the same mesophyll cells. As a highly infectious RNA virus, TRV rapidly activates plant defense pathways including PTGS, placing systemic tissues into an antiviral state. This in turn impedes the spread of CLCrV, a DNA virus that relies on nuclear entry and replicates relatively slowly. Consequently, the two viruses fail to act cooperatively within the same plant.

Subsequently, we demonstrated that tandem assembly of *GhCLA1* silencing fragments and sgRNA expression elements into the same virus vector could simultaneously silence the *GhCLA1* and edit the target gene (Figure 4). Although this strategy enables concurrent gene silencing and genome editing, the initial gene editing efficiency was relatively low. (Figure 5 and Supplementary Table S2). The insertion of exogenous DNA fragments into the viral genome can reduce the efficiency of the virus’s replication and spread in plants, particularly as the fragment length increases. Additionally, the inserted fragment may be lost or undergo mutations to improve the virus’s fitness [38,39]. This phenomenon is likely the primary cause of the low efficiency of gene editing observed in this experiment, as tandem assembly of silencing fragments and sgRNA expression elements increases viral DNA. Given that the sgRNA expression element was fixed and driven by the 330 bp AtU6-26 promoter, reducing the length of the silencing fragments could enhance the virus’s replication and spread, thus increasing the expression levels of sgRNA and potentially achieving more effective gene editing. To test whether shortened silencing fragments in tandem with sgRNAs could both efficiently silence and improve gene editing efficiency, we performed truncation cloning based on the 431 bp fragment that efficiently mediates *GhCLA1* silencing, obtained two shorter *GhCLA1* silencing fragments, and inserted them into viral vectors harboring *GhCLA1* sgRNA. (Supplementary Figure S6). We found that expression of the 282 bp *GhCLA1* fragment via TRV and CLCrV vectors failed to effectively suppress gene expression. We speculate that this fragment may lack key regions recognizable by DCL for efficient production of functional siRNAs, thus preventing efficient silencing of *GhCLA1*. However, a 368 bp fragment of *GhCLA1*, expressed via both TRV and CLCrV vectors, not only efficiently silenced *GhCLA1* but also improved the efficiency of *GhCLA1* gene editing (Figure 6 and Supplementary Table S3). As dynamic monitoring of viral accumulation levels was not performed for vectors carrying *GhCLA1* inserts of different lengths in this study, the observed improvement in gene editing efficiency may be associated with the reduced vector burden and enhanced systemic viral movement conferred by shorter inserts. This inference is based on indirect analyses of editing efficiency, and the underlying mechanism warrants further verification through quantitative detection of viral accumulation in future investigations. Furthermore, preferential amplification between the A and D subgenomes was detected in some plants in this study, and in some cases only a single subgenome could be effectively amplified. In contrast, the read ratio of the A to D subgenomes was approximately 1:1 in control plants (Cas9-OE plants not inoculated with viral vectors), indicating that such amplification bias was not caused by differences in primer specificity. We speculate that this preferential amplification may be related to the somatic chimerism of mutant individuals: small insertions or deletions at the target site lead to differences in GC content and secondary structure between the amplicons of the two subgenomes, thereby resulting in uneven amplification efficiency of the templates. This successful development of the simultaneous gene silencing and gene editing system in cotton lays a solid foundation for future experiments. First, this system overcomes the traditional paradigm in which gene silencing and genome editing depend on separate viral vectors, therefore simplifying experimental workflow and mitigating the risk of viral vector incompatibility. Second, this system provides a powerful technical tool for elucidating the interaction mechanism between plant antiviral genes and VIGE. In particular, antiviral genes in cotton can be selectively silenced by substituting the silencing fragment, thereby promoting viral systemic spread and accumulation and enhancing genome-editing efficiency. Finally, the design principles of this system are readily transferable to other genetically recalcitrant crops (e.g., wheat, soybean), thereby expanding the potential for broad application across diverse crop species.

However, several potential limitations and challenges remain for this system. One major challenge is how to simultaneously edit four copies of the target gene in cotton, an allotetraploid species (AADD genome), with at least one copy present in both the A and D. Another challenge is the limited cargo capacity of plant DNA and RNA viruses, which necessitates the use of stable transgenic Cas9-OE lines. Recent advances in the development of smaller nucleases, such as Cas12e, Cas12j and Cas12f (400–1000 amino acids) [40,41,42], offer promise for overcoming these constraints.

In this study, we established a technical framework that enables simultaneous gene silencing and editing in cotton using viral vectors, providing practical insights into the optimization of viral delivery systems.

## Data Availability

The data presented in this study are available in the article or the Supplementary Materials.

## Supplementary Materials

The following supporting information can be downloaded at: https://www.mdpi.com/article/10.3390/plants15081153/s1, Figure S1: PCR/RE detection of *GhCLA1* mutations in systemic leaves from Groups 1 and 3. Figure S2: Detection of TRV-V2 and CLCrV-B virus accumulation in systemic leaves from Group 1. Figure S3: DNA sequence of targeted editing of *GhCLA1* by *GhCLA1*-sgRNA. Figure S4: DNA sequence of targeted editing of *GhAGL16* by *GhAGL16*-sgRNA. Figure S5: Comparison of efficiency between TRV- and CLCrV-mediated simultaneous gene silencing and editing systems in cotton. Figure S6: Enzyme digestion of vectors containing *GhCLA1* silencing fragments of varying length in tandem with editing components for TRV- and CLCrV-mediated transient transformation. Figure S7: Phenotypic characterization and expression level assay of *GhCLA1* Silencing. Figure S8: DNA sequence of targeted editing of *GhCLA1* by TRV:*GhCLA1i*^368bp^-*GhCLA1*-sgRNA (Group 10) and CLCrV:*GhCLA1i*^368bp^-*GhCLA1*-sgRNA (Group 12). Table S1: Primer sequences used in this study. Table S2: High-throughput sequencing results for Groups 5–8. Table S3: High-throughput sequencing results for Groups 10, 12, TRV-*GhCLA1*-sgRNA and CLCrV-*GhCLA1*-sgRNA.
