# Supplementary material for: Virus-Mediated System for Simultaneous Gene Silencing and Genome Editing in Cotton
Source: Plants (Basel). 2026 Apr 9;15(8):1153. doi: 10.3390/plants15081153 (PMC13119147; doi:10.3390/plants15081153)
Supplement: Supplementary file 1 [file plants-15-01153-s001.zip › plants-4197872-supplementary.pdf]

Supplementary material

Virus-Mediated System for Simultaneous Gene Silencing and Genome Editing in Cotton

Yufeng Zheng [1,†], Lianjia Zhao [2,†], Yulin Tian [1], Jiahao Lin [3], Xiaodong Liu [1,4,*] and Jianfeng Lei [3,*]

1 Innovation Research Center for Efficient Genome Editing Technology and Precision Breeding Applications, College of Life Sciences, Xinjiang Agricultural University, Urumqi 830052, China; 18709006766@139.com (Y.Z.); ts117lin@foxmail.com (Y.T.)
2 Institute of Crop Research, Xinjiang Uyghur Autonomous Region Academy of Agricultural Sciences, Urumqi 830091, China; zlj4537163@163.com
3 Research Center of Cotton Engineering, College of Agronomy, Xinjiang Agricultural University, Ministry of Education, Urumqi 830052, China; linjiahao0628@163.com
4 College of Smart Agriculture, Xinjiang University, Urumqi 830046, China
* Correspondence: xiaodongliu75@aliyun.com (X.L.); kyleijianfeng@163.com (J.L.)
† These authors contributed equally to this work.

The following Supplementary material is available for this article:

Figure S1. PCR/RE detection of *GhCLA1* mutations in systemic leaves from Groups 1 and 3.

Figure S2. Detection of TRV-V2 and CLCrV-B virus accumulation in systemic leaves from Group 1.

Figure S3. DNA sequence of targeted editing of *GhCLA1* by *GhCLA1*-sgRNA.

Figure S4. DNA sequence of targeted editing of *GhAGL16* by *GhAGL16*-sgRNA.

Figure S5. Comparison of efficiency between TRV- and CLCrV-mediated simultaneous gene silencing and editing systems in cotton.

Figure S6. Enzyme digestion of vectors containing *GhCLA1* silencing fragments of varying length in tandem with editing components for TRV- and CLCrV-mediated transient transformation.

Figure S7. Phenotypic characterization and expression level assay of *GhCLA1* Silencing.

Figure S8. DNA sequence of targeted editing of *GhCLA1* by TRV:*GhCLA1i*^{368bp}-*GhCLA1*-sgRNA (Group 10) and CLCrV:*GhCLA1i*^{368bp}-*GhCLA1*-sgRNA (Group 12).

Table S1. Primer sequences used in this study.

Table S2. High-throughput sequencing results for Groups 5-8.

Table S3. High-throughput sequencing results for Groups 10, 12, TRV-*GhCLA1*-sgRNA and CLCrV-*GhCLA1*-sgRNA.

Figure S1. PCR/RE detection of *GhCLA1* mutations in systemic leaves from Groups 1 and 3.

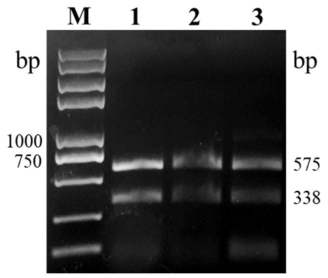

Lane 1: control; lane 2: Group 1; lane 3: Group 3.

Figure S2. Detection of TRV-V2 and CLCrV-B virus accumulation in systemic leaves from Group 1.

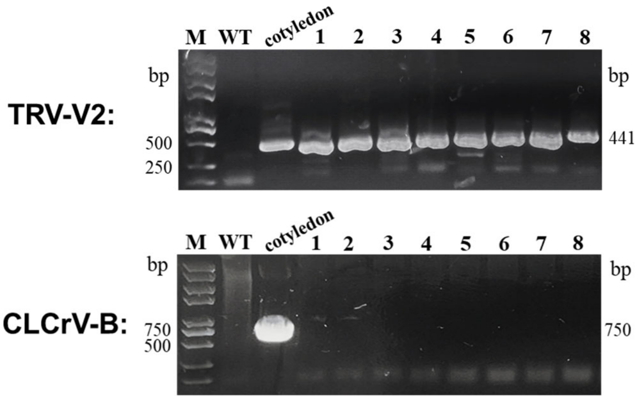

Figure S3. DNA sequence of targeted editing of *GhCLA1* by *GhCLA1*-sgRNA.

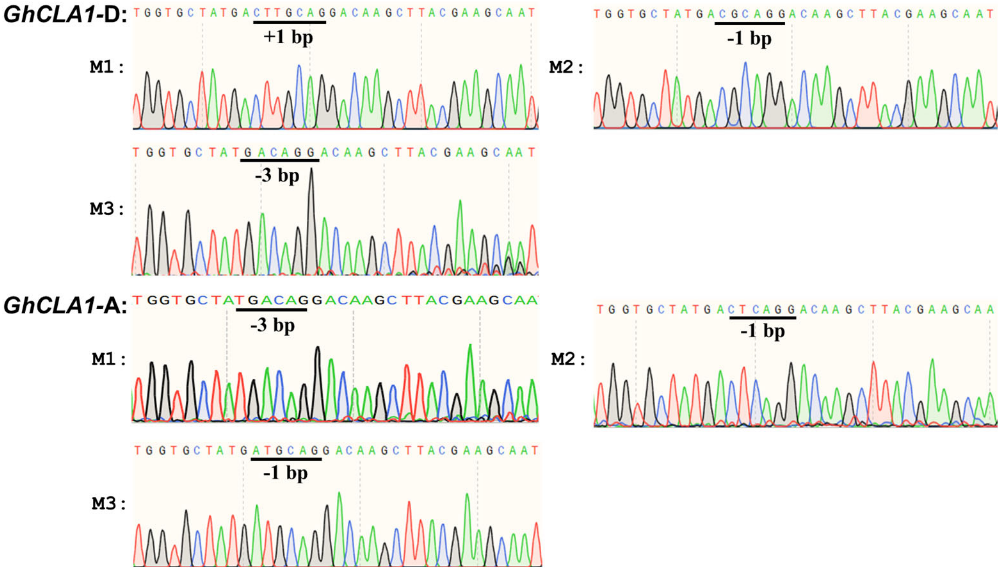

Figure S4. DNA sequence of targeted editing of *GhAGL16* by *GhAGL16*-sgRNA.

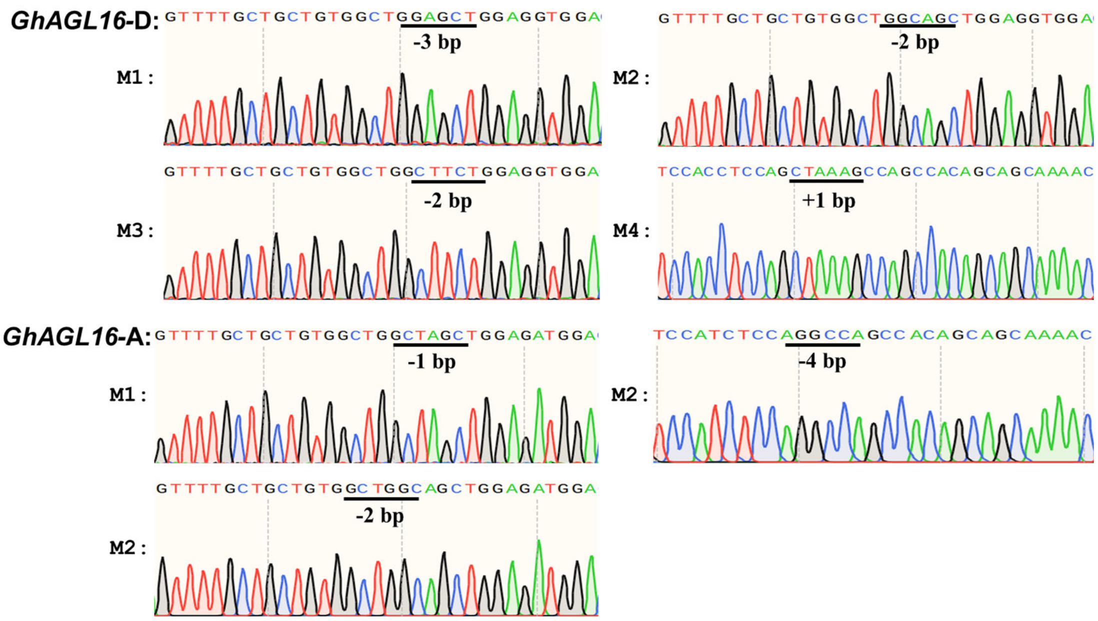

Figure S5. Comparison of efficiency between TRV- and CLCrV-mediated simultaneous gene silencing and editing systems in cotton.

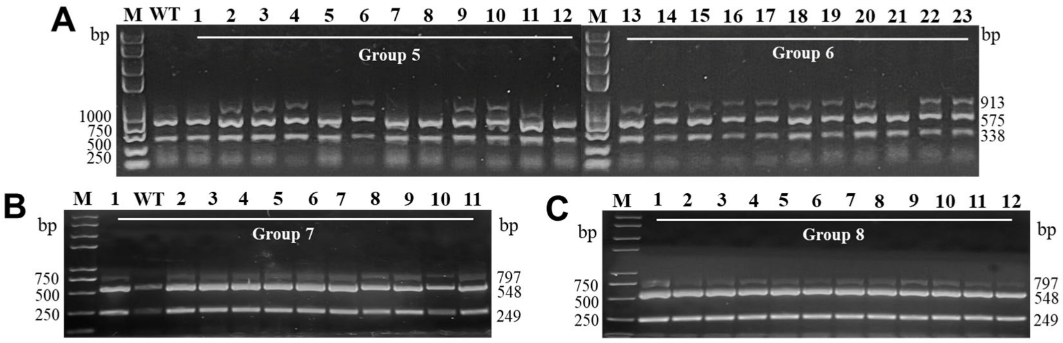

(A) PCR/RE mutation detection of *GhCLA1* in individual plants from Groups 5 and 6. WT serves as the control, lanes 1~12: Group 5 lines; lanes 13~ 23: Group 6 lines. **(B)** PCR/RE mutation detection of *GhAGL16* in individual plants from Group 7. WT serves as the control, lanes 1~11: Group 7 lines. **(C)** PCR/RE mutation detection of *GhAGL16* in single plants from Group 8, lanes 1~12: Group 8 lines.

Figure S6. Enzyme digestion of vectors containing *GhCLA1* silencing fragments of varying length in tandem with editing components for TRV- and CLCrV-mediated transient transformation.

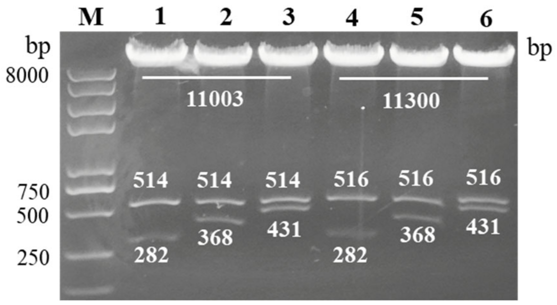

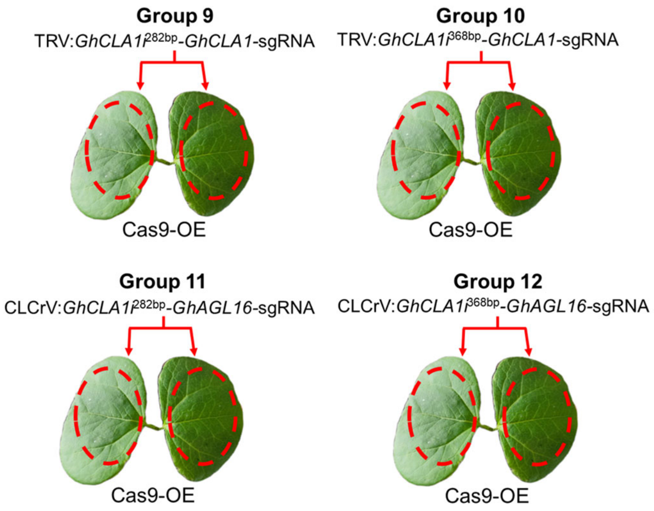

1. TRV:*GhCLA1i*^{282bp}-*GhCLA1*-sgRNA (Group 9); 2. TRV:*GhCLA1i*^{368bp}-*GhCLA1*-sgRNA (Group 10); 3. TRV:*GhCLA1i*^{431bp}-*GhCLA1*-sgRNA (Group 5); 4. CLCrV:*GhCLA1i*^{282bp}-*GhCLA1*-sgRNA (Group 11); 5. CLCrV:*GhCLA1i*^{368bp}-*GhCLA1*-sgRNA (Group 12); 6. CLCrV:*GhCLA1i*^{431bp}-*GhCLA1*-sgRNA (Group 6).

Figure S7. Phenotypic characterization and expression level assay of *GhCLA1* silencing.

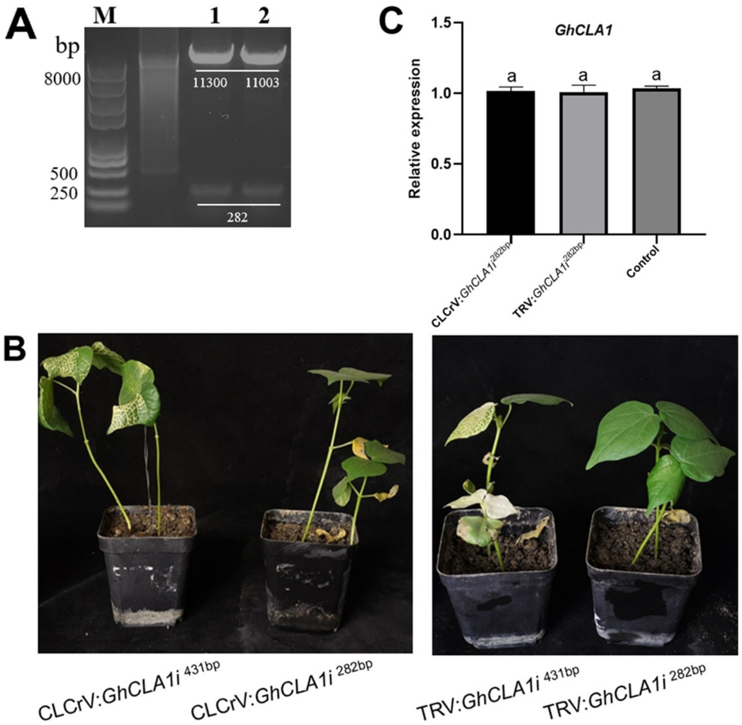

(A) Enzyme digestion identification of CLCrV and TRV viral vectors harboring 282 bp *GhCLA1* silencing fragments. 1. CLCrV:*GhCLA1i*^{282bp}; 2. TRV:*GhCLA1i*^{282bp}; **(B)** Inoculation of distinct experimental treatments on Cas9-OE cotton plants (n=12 plants per treatment). Fifteen to twenty days post inoculation, only the positive control plants exhibited distinct *GhCLA1* gene silencing phenotypes, while no such phenotype was observed in the remaining plants inoculated with the 282 bp *GhCLA1* silencing fragment. **(C)** Expression of *GhCLA1* in different experimental treatments (n=3). Lowercase letters indicate significant differences at the 0.05 level compared with the control.

Figure S8. DNA sequence of targeted editing of *GhCLA1* by TRV:*GhCLA1i*^{368bp}-*GhCLA1*-sgRNA

(Group 10) and CLCrV:*GhCLA1i*^{368bp}-*GhCLA1*-sgRNA (Group 12).

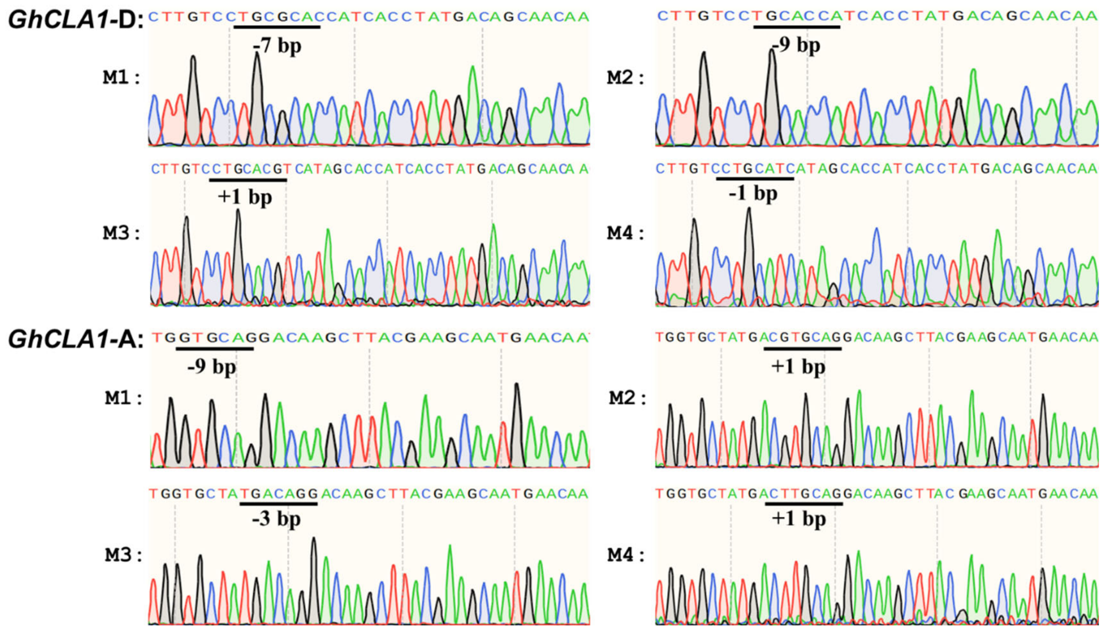

Table S1. Primer sequences used in this study.

Primer	Sequence (5'-3')	Application
*Eco*RI-*GhCLA1*(282)-*Bam*HIF:	GAATTCCTACCCAGTCTTACACTACA	
*Eco*RI-*GhCLA1*(282)-*Bam*HIR:	GGATCCTCTGCAGATCCACATCGTGT	Amplification of a 282 bp *GhCLA1* silencing
*Spe*I-*GhCLA1*(282)-*Pac*IF:	ACTAGTCTACCCAGTCTTACACTACA	fragments
*Spe*I-*GhCLA1*(282)-*Pac*IR:	TTAATTAATCTGCAGATCCACATCGTGT	
*Eco*RI-*GhCLA1*(368)-*Bam*HIF:	GAATTCGCCACAACATCGATGATTTAG	
*EcoR*I-GhCLA1(368)-*Bam*HIR:	GGATCCATGTTGTTCGGCTATCCCC	Amplification of a 368 bp *GhCLA1* silencing
*Spe*I-*GhCLA1*(368)-*Pac*IF:	ACTAGTGCCACAACATCGATGATTTAG	fragments
*Spe*I-*GhCLA1*(368)-*Pac*IR:	TTAATTAAATGTTGTTCGGCTATCCCC	
*Eco*RI-*GhCLA1*(431)-*Bam*HIF:	GAATTCCACAACATCGATGATTTAG	
*Eco*RI-*GhCLA1*(431)-*Bam*HIR:	GGATCCATGATGAGTAGATTGCAC	Amplification of a 431 bp *GhCLA1* silencing
*Spe*I-*GhCLA1*(431)-*Pac*IF:	ACTAGTCACAACATCGATGATTTAG	fragments
*Spe*I-*GhCLA1*(431)-*Pac*IR:	TTAATTAAATGATGAGTAGATTGCAC	
AtU6-*Bam*HIF:	GGATCCCTTGAACCGTAGCTTTCGTT	
AtU6-*Kpn*IR:	GGTACCATCGATACCGTCGACCTC	
AtU6-*Spe*IF:	ACTAGTCTTGAACCGTAGCTTTCGTT	Modification of AtU6-26::sgRNA vector
AtU6-*Pac*IR:	TTAATTAAATCGATACCGTCGACCTC	
AtU6-*Pac*IF:	TTAATTAACTTGAACCGTAGCTTTCGTT	
AtU6-*Avr*IIR:	CCTAGGATCGATACCGTCGACCTC	
M-*GhCLA1*F:	GCAGGCTTGGGTATTAATCC	Amplification of DNA fragments flanking
M-*GhCLA1*R:	GGCTTATTCTGTATGTTACCG	*GhCLA1* target from cotton A, D genome
M-*GhAGL16*F:	TCCGTATGAAGAAGGTTTGAAAATT	Amplification of DNA fragments flanking
M-*GhAGL16*R:	GAGAAGCATAACTTTTGGAACC	*GhAGL16* target from cotton A, D genome
TRV-V2F:	AATGTCATACCACTTGTCCGC	TRV-V2 virus detection
TRV-V2R:	AAGGTCCTGCACTTTGAACG	

CLCrV-BF:	ATGTACAGTTTAAAGAGTAGACG	CLCrV-B virus detection
CLCrV-BR:	ATTATCCAATATAATCAAGGTCATAC	
Q-*GhCLA1*F:	ATGCATCACTATCAGAAGGAG	qPCR analysis of *GhCLA1* mRNA expression
Q-*GhCLA1*R:	CACATCAGACCGCAGTTCTT	
Q-*GhUBQ7*F:	GAAGGCATTCCACCTGACCAAC	qPCR analysis of *GhUBQ7* mRNA expression
Q-*GhUBQ7*R:	CTTGACCTTCTTCTTCTTGTGCTTG	
HiTom-*GhAGL16*F:	GGAGTGAGTACGGTGTGCCAAGGGATGTCAATGGTGCA	Detection of *GhAGL16* gene editing efficiency
HiTom-*GhAGL16*R:	GAGTTGGATGCTGGATGGTGGAGATCAGTTTGTGGCAA	by high-throughput sequencing
HiTom-*GhCLA1*F:	GGAGTGAGTACGGTGTGCGAAGGGATCTGAAAGGTGAA	Detection of *GhCLA1* gene editing efficiency
HiTom-*GhCLA1*R:	GAGTTGGATGCTGGATGGCTCTAAGAGGCCTGTTTGAT	by high-throughput sequencing

Table S2. High-throughput sequencing results for Groups 5-8.

1. High-throughput sequencing results for Groups 5.

Genome	Plant number	Number of sample reads	Mutation ratio	Mutation type	Mutations in the sequence	Mutation site
Subgroup A	WT	1029	100.00%	WT	-	GGTGATGGTGCTATGACTGCAGG
	#2	903	91.96%	WT	-	GGTGATGGTGCTATGACTGCAGG
		62	6.31%	6D	CTATGA	GGTGATGGTG------CTGCAGG
		17	1.73%	1I	T	GGTGATGGTGCTATGACTTGCAGG
	#4	973	95.02%	WT	-	GGTGATGGTGCTATGACTGCAGG
		33	3.22%	2D	AC	GGTGATGGTGCTATG--TGCAGG
		18	1.76%	1D	T	GGTGATGGTGCTATGAC-GCAGG
	#6	1334	96.32%	WT	-	GGTGATGGTGCTATGACTGCAGG
		23	1.66%	1I	A	GGTGATGGTGCTATGACATGCAGG
		17	1.23%	8D	GCTATGAC	GGTGATGGT--------TGCAGG
		11	0.79%	4D	TGAC	GGTGATGGTGCTA----TGCAGG
	#10	775	93.15%	WT	-	GGTGATGGTGCTATGACTGCAGG
		31	3.73%	SNP	C->T	GGTGATGGTGCTATGACTGTAGG
		26	3.13%	1D	C	GGTGATGGTGCTATGA-TGCAGG
	#11	914	94.62%	WT	-	GGTGATGGTGCTATGACTGCAGG
		52	5.38%	3D,2D	TAT,AC	GGTGATGGTGC---G--TGCAGG
Subgroup D	WT	998	100.00%	WT	-	GGTGATGGTGCTATGACTGCAGG
	#2	922	92.02%	WT	-	GGTGATGGTGCTATGACTGCAGG
		52	5.19%	1I	T	GGTGATGGTGCTATGACTTGCAGG
		28	2.79%	6D	CTATGA	GGTGATGGTG------CTGCAGG
	#4	1223	94.66%	WT	-	GGTGATGGTGCTATGACTGCAGG
		69	5.34%	1I	T	GGTGATGGTGCTATGACTTGCAGG
	#3	936	96.40%	WT	-	GGTGATGGTGCTATGACTGCAGG
		35	3.60%	1D	T	GGTGATGGTGCTATGAC-GCAGG
	#6	1032	90.93%	WT	-	GGTGATGGTGCTATGACTGCAGG
		54	4.76%	2D	AC	GGTGATGGTGCTATG--TGCAGG
		49	4.32%	1I	A	GGTGATGGTGCTATGACATGCAGG

	#9	974	94.66%	WT	-	GGTGATGGTGCTATGACTGCAGG
		55	5.34%	6D	CTATGA	GGTGATGGTG------CTGCAGG
	#10	1384	88.72%	WT	-	GGTGATGGTGCTATGACTGCAGG
		112	7.18%	1I	T	GGTGATGGTGCTATGACTTGCAGG
		64	4.10%	3D,2D	TAT,AC	GGTGATGGTGC---G--TGCAGG
	#11	1119	95.23%	WT	-	GGTGATGGTGCTATGACTGCAGG
		35	2.98%	1I	T	GGTGATGGTGCTATGACTTGCAGG
		21	1.79%	1D	T	GGTGATGGTGCTATGAC-GCAGG

WT is the control; SNP indicates base substitution; D indicates base deletion; I indicates base insertion

2. High-throughput sequencing results for Groups 6.

Genome	Plant number	Number of sample reads	Mutation ratio	Mutation type	Mutations in the sequence	Mutation site
Subgroup A	WT	1029	100.00%	WT	-	GGTGATGGTGCTATGACTGCAGG
	#1	1002	96.44%	WT	-	GGTGATGGTGCTATGACTGCAGG
		37	3.56%	2D	AC	GGTGATGGTGCTATG--TGCAGG
	#2	1146	93.25%	WT	-	GGTGATGGTGCTATGACTGCAGG
		45	3.66%	1D	C	GGTGATGGTGCTATGA-TGCAGG
		27	2.20%	4D	TGAC	GGTGATGGTGCTA----TGCAGG
		11	0.90%	1I	C	GGTGATGGTGCTATGACCTGCAGG
	#5	1001	95.15%	WT	-	GGTGATGGTGCTATGACTGCAGG
		51	4.85%	1D	T	GGTGATGGTGCTATGAC-GCAGG
	#3	1334	94.95%	WT	-	GGTGATGGTGCTATGACTGCAGG
		43	3.06%	1I	A	GGTGATGGTGCTATGACATGCAGG
		17	1.21%	8D	GCTATGAC	GGTGATGGT--------TGCAGG
		11	0.78%	4D	TGAC	GGTGATGGTGCTA----TGCAGG
	#7	1201	97.25%	WT	-	GGTGATGGTGCTATGACTGCAGG
		34	2.75%	1D	C	GGTGATGGTGCTATGA-TGCAGG
	#6	911	84.35%	WT	-	GGTGATGGTGCTATGACTGCAGG
		112	10.37%	1I	T	GGTGATGGTGCTATGACTTGCAGG
		57	5.28%	1D	C	GGTGATGGTGCTATGA-TGCAGG
	#8	883	87.69%	WT	-	GGTGATGGTGCTATGACTGCAGG
		75	7.45%	1I	A	GGTGATGGTGCTATGACATGCAGG
		49	4.87%	6D	CTATGA	GGTGATGGTG------CTGCAGG
	#11	1002	95.70%	WT	-	GGTGATGGTGCTATGACTGCAGG
		31	2.96%	3D,2D	TAT,AC	GGTGATGGTGC---G--TGCAGG
		14	1.34%	1I	T	GGTGATGGTGCTATGACTTGCAGG
Subgroup D	WT	998	100.00%	WT	-	GGTGATGGTGCTATGACTGCAGG
	#1	1453	93.38%	WT	-	GGTGATGGTGCTATGACTGCAGG
		78	5.01%	1I	T	GGTGATGGTGCTATGACTTGCAGG
		25	1.61%	1D	T	GGTGATGGTGCTATGAC-GCAGG
	#2	1176	96.95%	WT	-	GGTGATGGTGCTATGACTGCAGG

		37	3.05%	4D	TGAC	GGTGATGGTGCTA----TGCAGG
	#4	973	91.10%	WT	-	GGTGATGGTGCTATGACTGCAGG
		63	5.90%	1I	T	GGTGATGGTGCTATGACTTGCAGG
		32	3.00%	1D	T	GGTGATGGTGCTATGAC-GCAGG
	#7	1159	96.83%	WT	-	GGTGATGGTGCTATGACTGCAGG
		38	3.17%	1D	C	GGTGATGGTGCTATGA-TGCAGG
	#6	1293	92.95%	WT	-	GGTGATGGTGCTATGACTGCAGG
		46	3.31%	4D	TGAC	GGTGATGGTGCTA----TGCAGG
		37	2.66%	1I	T	GGTGATGGTGCTATGACTTGCAGG
		15	1.08%	1D	T	GGTGATGGTGCTATGAC-GCAGG
	#11	831	97.08%	WT	-	GGTGATGGTGCTATGACTGCAGG
		25	2.92%	1I	A	GGTGATGGTGCTATGACATGCAGG
	#10	1019	93.74%	WT	-	GGTGATGGTGCTATGACTGCAGG
		57	5.24%	1D	T	GGTGATGGTGCTATGAC-GCAGG
		11	1.01%	1D	C	GGTGATGGTGCTATGA-TGCAGG

WT is the control; SNP indicates base substitution; D indicates base deletion; I indicates base insertion

3. High-throughput sequencing results for Groups 7.

Genome	Plant number	Number of sample reads	Mutation ratio	Mutation type	Mutations in the sequence	Mutation site
Subgroup A	WT	1212	100.00%	WT	-	CCAGCTAAGCCAGCCACAGCAGC
	#1	1162	94.70%	WT	-	CCAGCTAAGCCAGCCACAGCAGC
		65	5.30%	SNP	G->A	CCAACTAAGCCAGCCACAGCAGC
	#4	1091	96.81%	WT	-	CCAGCTAAGCCAGCCACAGCAGC
		36	3.19%	4D	AGCC	CCAGCTA----AGCCACAGCAGC
	#5	1015	94.33%	WT	-	CCAGCTAAGCCAGCCACAGCAGC
		44	4.09%	2D	AA	CCAGCT--GCCAGCCACAGCAGC
		17	1.58%	3D	AAG	CCAGCT---CCAGCCACAGCAGC
	#9	901	96.67%	WT	-	CCAGCTAAGCCAGCCACAGCAGC
		31	3.33%	2D	AA	CCAGCT--GCCAGCCACAGCAGC
	#11	777	94.87%	WT	-	CCAGCTAAGCCAGCCACAGCAGC
		42	5.13%	3D	AAG	CCAGCT---CCAGCCACAGCAGC
Subgroup D	WT	1625	100.00%	WT	-	CCAGCTAAGCCAGCCACAGCAGC
	#2	1811	97.89%	WT	-	CCAGCTAAGCCAGCCACAGCAGC
		39	2.11%	1I	A	CCAGCTAAAGCCAGCCACAGCAGC
	#3	1556	96.59%	WT	-	CCAGCTAAGCCAGCCACAGCAGC
		37	2.30%	SNP	A->G	CCGGCTAAGCCAGCCACAGCAGC
		18	1.12%	SNP	C->T	CCAGCTAAGTCAGCCACAGCAGC
	#4	1765	96.13%	WT	-	CCAGCTAAGCCAGCCACAGCAGC
		36	1.96%	3D	AAG	CCAGCT---CCAGCCACAGCAGC
		35	1.91%	1I	A	CCAGCTAAAGCCAGCCACAGCAGC

	#5	1715	94.86%	WT	-	CCAGCTAAGCCAGCCACAGCAGC
		93	5.14%	3D	AAG	CCAGCT---CCAGCCACAGCAGC
	#6	1951	96.44%	WT	-	CCAGCTAAGCCAGCCACAGCAGC
		38	1.88%	1D	A	CCAGCT-AGCCAGCCACAGCAGC
		34	1.68%	1I	A	CCAGCTAAAGCCAGCCACAGCAGC
	#7	1594	96.78%	WT	-	CCAGCTAAGCCAGCCACAGCAGC
		53	3.22%	1I	A	CCAGCTAAAGCCAGCCACAGCAGC
	#8	1624	84.85%	WT	-	CCAGCTAAGCCAGCCACAGCAGC
		269	14.05%	1I	A	CCAGCTAAAGCCAGCCACAGCAGC
		21	1.10%	3D	AAG	CCAGCT---CCAGCCACAGCAGC
	#9	1502	98.17%	WT	-	CCAGCTAAGCCAGCCACAGCAGC
		28	1.83%	3D	AAG	CCAGCT---CCAGCCACAGCAGC
	#10	1506	97.98%	WT	-	CCAGCTAAGCCAGCCACAGCAGC
		31	2.02%	6D	CTAAGC	CCAG------CAGCCACAGCAGC

WT is the control; SNP indicates base substitution; D indicates base deletion; I indicates base insertion

4. High-throughput sequencing results for Groups 8.

Genome	Plant number	Number of sample reads	Mutation ratio	Mutation type	Mutations in the sequence	Mutation site
Subgroup A	WT	1212	100.00%	WT	-	CCAGCTAAGCCAGCCACAGCAGC
	#2	1050	96.60%	WT	-	CCAGCTAAGCCAGCCACAGCAGC
		37	3.40%	SNP	A->G	CCAGCTAAGCCGGCCACAGCAGC
	#3	1249	95.64%	WT	-	CCAGCTAAGCCAGCCACAGCAGC
		40	3.06%	2D	AA	CCAGCT--GCCAGCCACAGCAGC
		17	1.30%	SNP	A->G	CCAGCTAAGCCAGCCACGGCAGC
	#6	1029	97.72%	WT	-	CCAGCTAAGCCAGCCACAGCAGC
		24	2.28%	2D	AA	CCAGCT--GCCAGCCACAGCAGC
	#7	994	93.69%	WT	-	CCAGCTAAGCCAGCCACAGCAGC
		67	6.31%	1D	A	CCAGCT-AGCCAGCCACAGCAGC
	#9	1257	96.54%	WT	-	CCAGCTAAGCCAGCCACAGCAGC
		45	3.46%	2D	AA	CCAGCT--GCCAGCCACAGCAGC
	#10	1017	93.22%	WT	-	CCAGCTAAGCCAGCCACAGCAGC
		43	3.94%	3D	AAG	CCAGCT---CCAGCCACAGCAGC
		31	2.84%	1I	A	CCAGCTAAAGCCAGCCACAGCAGC
Subgroup D	WT	1625	100.00%	WT	-	CCAGCTAAGCCAGCCACAGCAGC
	#1	1576	94.48%	WT	-	CCAGCTAAGCCAGCCACAGCAGC
		51	3.06%	1I	A	CCAGCTAAAGCCAGCCACAGCAGC
		41	2.46%	SNP	T->C	CCAGCCAAGCCAGCCACAGCAGC
	#3	1737	96.45%	WT	-	CCAGCTAAGCCAGCCACAGCAGC
		64	3.55%	3D	AAG	CCAGCT---CCAGCCACAGCAGC
	#4	1170	94.51%	WT	-	CCAGCTAAGCCAGCCACAGCAGC

	35	2.83%	3D	AAG	CCAGCT---CCAGCCACAGCAGC
	33	2.67%	SNP	C->T	CCAGCTAAGCCAGCTACAGCAGC
#5	1603	96.86%	WT	-	CCAGCTAAGCCAGCCACAGCAGC
	52	3.14%	3D	AAG	CCAGCT---CCAGCCACAGCAGC
#8	1503	96.97%	WT	-	CCAGCTAAGCCAGCCACAGCAGC
	47	3.03%	2D	AA	CCAGCT--GCCAGCCACAGCAGC
#9	1495	98.81%	WT	-	CCAGCTAAGCCAGCCACAGCAGC
	18	1.19%	3D	AAG	CCAGCT---CCAGCCACAGCAGC
#10	1405	89.72%	WT	-	CCAGCTAAGCCAGCCACAGCAGC
	101	6.45%	1D	A	CCAGCT-AGCCAGCCACAGCAGC
	60	3.83%	3D	AAG	CCAGCT---CCAGCCACAGCAGC
#11	1624	93.87%	WT	-	CCAGCTAAGCCAGCCACAGCAGC
	85	4.91%	2D	AA	CCAGCT--GCCAGCCACAGCAGC
	21	1.21%	1D	A	CCAGCT-AGCCAGCCACAGCAGC
#12	1482	96.48%	WT	-	CCAGCTAAGCCAGCCACAGCAGC
	54	3.52%	2D	AA	CCAGCT--GCCAGCCACAGCAGC

WT is the control; SNP indicates base substitution; D indicates base deletion; I indicates base insertion

Table S3. High-throughput sequencing results for Groups 10, 12, TRV-*GhCLA1*-sgRNA and CLCrV-*GhCLA1*-sgRNA.

1. High-throughput sequencing results for Groups 10.

Genome	Plant number	Number of sample reads	Mutation ratio	Mutation type	Mutations in the sequence	Mutation site
Subgroup A	WT	1367	100.00%	WT	-	GGTGATGGTGCTATGACTGCAGG
	#1	833	76.35%	WT	-	GGTGATGGTGCTATGACTGCAGG
		153	14.02%	1D	C	GGTGATGGTGCTATGA-TGCAGG
		67	6.14%	2D	CT	GGTGATGGTGCTATGA--GCAGG
		38	3.48%	3I	GAA	GGTGATGGTGCTATGACGAATGCAGG
	#2	742	71.00%	WT	-	GGTGATGGTGCTATGACTGCAGG
		219	20.96%	2D	CT	GGTGATGGTGCTATGA--GCAGG
		84	8.04%	3D	TGA	GGTGATGGTGCTA---CTGCAGG
	#4	973	78.85%	WT	-	GGTGATGGTGCTATGACTGCAGG
		133	10.78%	2D	AC	GGTGATGGTGCTATG--TGCAGG
		97	7.86%	2D	CT	GGTGATGGTGCTATGA--GCAGG
		31	2.51%	1I	A	GGTGATGGTGCTATGACATGCAGG
	#5	882	83.92%	WT	-	GGTGATGGTGCTATGACTGCAGG
		67	6.37%	3D	TGA	GGTGATGGTGCTA---CTGCAGG
		59	5.61%	2D	AC	GGTGATGGTGCTATG--TGCAGG
		43	4.09%	5D	ATGAC	GGTGATGGTGCT-----TGCAGG
	#6	761	82.72%	WT	-	GGTGATGGTGCTATGACTGCAGG

		114	12.39%	1I	T	GGTGATGGTGCTATGACTTGCAGG
		45	4.89%	6D	CTATGA	GGTGATGGTG------CTGCAGG
	#8	775	75.54%	WT	-	GGTGATGGTGCTATGACTGCAGG
		157	15.30%	1D	T	GGTGATGGTGCTATGAC-GCAGG
		71	6.92%	SNP	C->T	GGTGATGGTGCTATGACTGTAGG
		23	2.24%	1D	C	GGTGATGGTGCTATGA-TGCAGG
	#10	1032	87.98%	WT	-	GGTGATGGTGCTATGACTGCAGG
		73	6.22%	2D	AC	GGTGATGGTGCTATG--TGCAGG
		68	5.80%	1D	C	GGTGATGGTGCTATGA-TGCAGG
Subgroup D	WT	1135	100.00%	WT	-	GGTGATGGTGCTATGACTGCAGG
	#1	887	80.49%	WT	-	GGTGATGGTGCTATGACTGCAGG
		93	8.44%	4D	TGAC	GGTGATGGTGCTA----TGCAGG
		77	6.99%	3D	TGA	GGTGATGGTGCTA---CTGCAGG
		45	4.08%	1D	T	GGTGATGGTGCTATGAC-GCAGG
	#2	871	83.03%	WT	-	GGTGATGGTGCTATGACTGCAGG
		113	10.77%	1I	A	GGTGATGGTGCTATGACATGCAGG
		65	6.20%	2D	CT	GGTGATGGTGCTATGA--GCAGG
	#3	1041	84.43%	WT	-	GGTGATGGTGCTATGACTGCAGG
		102	8.27%	1D	T	GGTGATGGTGCTATGAC-GCAGG
		73	5.92%	9D	TATGACTGC	GGTGATGGTGC---------AGG
		17	1.38%	4D	ACTG	GGTGATGGTGCTATG----CAGG
	#4	719	78.24%	WT	-	GGTGATGGTGCTATGACTGCAGG
		97	10.55%	6D	CTATGA	GGTGATGGTG------CTGCAGG
		64	6.96%	1I	A	GGTGATGGTGCTATGACATGCAGG
		39	4.24%	1D	T	GGTGATGGTGCTATGAC-GCAGG
	#5	1078	92.61%	WT	-	GGTGATGGTGCTATGACTGCAGG
		65	5.58%	1D	C	GGTGATGGTGCTATGA-TGCAGG
		21	1.80%	9D	TATGACTGC	GGTGATGGTGC---------AGG
	#6	691	74.22%	WT	-	GGTGATGGTGCTATGACTGCAGG
		109	11.71%	2D	CT	GGTGATGGTGCTATGA--GCAGG
		74	7.95%	1D	T	GGTGATGGTGCTATGAC-GCAGG
		35	3.76%	1I	A	GGTGATGGTGCTATGACATGCAGG
		22	2.36%	3I	GAA	GGTGATGGTGCTATGACGAATGCAGG
	#7	1143	83.98%	WT	-	GGTGATGGTGCTATGACTGCAGG
		193	14.18%	1D	T	GGTGATGGTGCTATGAC-GCAGG
		25	1.84%	4D	TGAC	GGTGATGGTGCTA----TGCAGG
	#8	959	82.60%	WT	-	GGTGATGGTGCTATGACTGCAGG
		104	8.96%	1D	C	GGTGATGGTGCTATGA-TGCAGG
		67	5.77%	2D	AC	GGTGATGGTGCTATG--TGCAGG
		31	2.67%	5D	ATGAC	GGTGATGGTGCT-----TGCAGG
	#9	743	64.00%	WT	-	GGTGATGGTGCTATGACTGCAGG

		174	14.99%	2D	CT	GGTGATGGTGCTATGA--GCAGG
		135	11.63%	3D	TGA	GGTGATGGTGCTA---CTGCAGG
		66	5.68%	2D	AC	GGTGATGGTGCTATG--TGCAGG
		43	3.70%	1D	T	GGTGATGGTGCTATGAC-GCAGG
	#10	991	94.65%	WT	-	GGTGATGGTGCTATGACTGCAGG
		35	3.34%	1I	T	GGTGATGGTGCTATGACTTGCAGG
		21	2.01%	1D	C	GGTGATGGTGCTATGA-TGCAGG

WT is the control; SNP indicates base substitution; D indicates base deletion; I indicates base insertion

2. High-throughput sequencing results for Groups 12.

Genome	Plant number	Number of sample reads	Mutation ratio	Mutation type	Mutations in the sequence	Mutation site
Subgroup A	WT	1367	100.00%	WT	-	GGTGATGGTGCTATGACTGCAGG
	#1	1094	86.55%	WT	-	GGTGATGGTGCTATGACTGCAGG
		93	7.36%	2D	CT	GGTGATGGTGCTATGA--GCAGG
		77	6.09%	3D	TGA	GGTGATGGTGCTA---CTGCAGG
		15	1.19%	1D	C	GGTGATGGTGCTATGA-TGCAGG
	#2	912	81.28%	WT	-	GGTGATGGTGCTATGACTGCAGG
		83	7.40%	1D	T	GGTGATGGTGCTATGAC-GCAGG
		76	6.77%	4D	TGAC	GGTGATGGTGCTA----TGCAGG
		51	4.55%	1I	T	GGTGATGGTGCTATGACTTGCAGG
	#3	1003	91.02%	WT	-	GGTGATGGTGCTATGACTGCAGG
		45	4.08%	3D	TGA	GGTGATGGTGCTA---CTGCAGG
		33	2.99%	2D	AC	GGTGATGGTGCTATG--TGCAGG
		21	1.91%	1D	C	GGTGATGGTGCTATGA-TGCAGG
	#4	975	84.78%	WT	-	GGTGATGGTGCTATGACTGCAGG
		113	9.83%	3D	TGA	GGTGATGGTGCTA---CTGCAGG
		62	5.39%	1I	A	GGTGATGGTGCTATGACATGCAGG
	#6	786	71.52%	WT	-	GGTGATGGTGCTATGACTGCAGG
		217	19.75%	2D	AC	GGTGATGGTGCTATG--TGCAGG
		73	6.64%	5D	ATGAC	GGTGATGGTGCT-----TGCAGG
		23	2.09%	1D	C	GGTGATGGTGCTATGA-TGCAGG
	#7	1101	81.19%	WT	-	GGTGATGGTGCTATGACTGCAGG
		157	11.58%	1I	T	GGTGATGGTGCTATGACTTGCAGG
		98	7.23%	6D	CTATGA	GGTGATGGTG------CTGCAGG
	#9	889	82.85%	WT	-	GGTGATGGTGCTATGACTGCAGG
		96	8.95%	1D	T	GGTGATGGTGCTATGAC-GCAGG
		65	6.06%	4D	ACTG	GGTGATGGTGCTATG----CAGG
		23	2.14%	1D	C	GGTGATGGTGCTATGA-TGCAGG
	#10	943	91.38%	WT	-	GGTGATGGTGCTATGACTGCAGG
		61	5.91%	1I	T	GGTGATGGTGCTATGACTTGCAGG
		28	2.71%	4D	TGAC	GGTGATGGTGCTA----TGCAGG

	WT	1135	100.00%	WT	-	GGTGATGGTGCTATGACTGCAGG
	#1	1100	87.65%	WT	-	GGTGATGGTGCTATGACTGCAGG
		93	7.41%	1D	C	GGTGATGGTGCTATGA-TGCAGG
		62	4.94%	3D	GAC	GGTGATGGTGCTAT---TGCAGG
	#2	901	85.00%	WT	-	GGTGATGGTGCTATGACTGCAGG
		126	11.89%	2D	AC	GGTGATGGTGCTATG--TGCAGG
		33	3.11%	2D	CT	GGTGATGGTGCTATGA--GCAGG
	#3	1223	84.87%	WT	-	GGTGATGGTGCTATGACTGCAGG
		79	5.48%	2D	AC	GGTGATGGTGCTATG--TGCAGG
		67	4.65%	1I	T	GGTGATGGTGCTATGACTTGCAGG
		33	2.29%	1D	T	GGTGATGGTGCTATGAC-GCAGG
		28	1.94%	6D	CTATGA	GGTGATGGTG------CTGCAGG
		11	0.76%	3D	TGA	GGTGATGGTGCTA---CTGCAGG
	#4	998	88.24%	WT	-	GGTGATGGTGCTATGACTGCAGG
		101	8.93%	1D	T	GGTGATGGTGCTATGAC-GCAGG
		32	2.83%	1I	A	GGTGATGGTGCTATGACATGCAGG
Subgroup D	#5	1119	80.33%	WT	-	GGTGATGGTGCTATGACTGCAGG
		175	12.56%	3D	TGA	GGTGATGGTGCTA---CTGCAGG
		82	5.89%	1D	T	GGTGATGGTGCTATGAC-GCAGG
		17	1.22%	1I	T	GGTGATGGTGCTATGACTTGCAGG
	#6	879	78.83%	WT	-	GGTGATGGTGCTATGACTGCAGG
		133	11.93%	2D	CT	GGTGATGGTGCTATGA--GCAGG
		66	5.92%	4D	ACTG	GGTGATGGTGCTATG----CAGG
		37	3.32%	1D	T	GGTGATGGTGCTATGAC-GCAGG
	#8	1009	79.70%	WT	-	GGTGATGGTGCTATGACTGCAGG
		128	10.11%	5D	ATGAC	GGTGATGGTGCT-----TGCAGG
		84	6.64%	1I	A	GGTGATGGTGCTATGACATGCAGG
		45	3.55%	2D	AC	GGTGATGGTGCTATG--TGCAGG
	#9	1075	79.04%	WT	-	GGTGATGGTGCTATGACTGCAGG
		156	11.47%	2D	AC	GGTGATGGTGCTATG--TGCAGG
		96	7.06%	3D	TGA	GGTGATGGTGCTA---CTGCAGG
		33	2.43%	1D	T	GGTGATGGTGCTATGAC-GCAGG
	#10	887	86.96%	WT	-	GGTGATGGTGCTATGACTGCAGG
		87	8.53%	3D	TGA	GGTGATGGTGCTA---CTGCAGG
		46	4.51%	8D	GCTATGAC	GGTGATGGT--------TGCAGG

WT is the control; SNP indicates base substitution; D indicates base deletion; I indicates base insertion

3. High-throughput sequencing results for TRV-*GhCLA1*-sgRNA.

Genome	Plant number	Number of sample reads	Mutation ratio	Mutation type	Mutations in the sequence	Mutation site

	WT	1422	100.00%	WT	-	GGTGATGGTGCTATGACTGCAGG
	#1	986	73.86%	WT	-	GGTGATGGTGCTATGACTGCAGG
		185	13.86%	1I	T	GGTGATGGTGCTATGACTTGCAGG
		89	6.67%	1I	G	GGTGATGGTGCTATGACGTGCAGG
		75	5.62%	1D	T	GGTGATGGTGCTATGAC-GCAGG
	#2	1329	75.30%	WT	-	GGTGATGGTGCTATGACTGCAGG
		203	11.50%	1D	T	GGTGATGGTGCTATGAC-GCAGG
		89	5.04%	1I	G	GGTGATGGTGCTATGACGTGCAGG
		57	3.23%	1D	C	GGTGATGGTGCTATGA-TGCAGG
		39	2.21%	6D	ATGACT	GGTGATGGTGCT------GCAGG
		25	1.42%	3D	GAC	GGTGATGGTGCTAT---TGCAGG
		23	1.30%	5D	ATGAC	GGTGATGGTGCT-----TGCAGG
	#3	866	57.12%	WT	-	GGTGATGGTGCTATGACTGCAGG
		351	23.15%	1I	T	GGTGATGGTGCTATGACTTGCAGG
		173	11.41%	1D	T	GGTGATGGTGCTATGAC-GCAGG
		77	5.08%	6D	ATGACT	GGTGATGGTGCT------GCAGG
		33	2.18%	1I	A	GGTGATGGTGCTATGACATGCAGG
		16	1.06%	4D	ACTG	GGTGATGGTGCTATG----CAGG
Subgroup A	#4	1143	78.61%	WT	-	GGTGATGGTGCTATGACTGCAGG
		109	7.50%	1I	T	GGTGATGGTGCTATGACTTGCAGG
		56	3.85%	1D	T	GGTGATGGTGCTATGAC-GCAGG
		42	2.89%	6D	ATGACT	GGTGATGGTGCT------GCAGG
		33	2.27%	4D	ACTG	GGTGATGGTGCTATG----CAGG
		29	1.99%	1I	C	GGTGATGGTGCTATGACCTGCAGG
		24	1.65%	1I	A	GGTGATGGTGCTATGACATGCAGG
		18	1.24%	2D	AC	GGTGATGGTGCTATG--TGCAGG
	#5	1229	92.13%	WT	-	GGTGATGGTGCTATGACTGCAGG
		74	5.55%	1I	A	GGTGATGGTGCTATGACATGCAGG
		31	2.32%	1D	T	GGTGATGGTGCTATGAC-GCAGG
	#6	994	62.16%	WT	-	GGTGATGGTGCTATGACTGCAGG
		236	14.76%	1I	T	GGTGATGGTGCTATGACTGTAGG
		182	11.38%	6D	ATGACT	GGTGATGGTGCT------GCAGG
		93	5.82%	1D	T	GGTGATGGTGCTATGAC-GCAGG
		35	2.19%	1D	C	GGTGATGGTGCTATGA-TGCAGG
		22	1.38%	3D	GAC	GGTGATGGTGCTAT---TGCAGG
		19	1.19%	1I	G	GGTGATGGTGCTATGACGTGCAGG
		18	1.13%	1I	A	GGTGATGGTGCTATGACATGCAGG
	#7	1741	95.66%	WT	-	GGTGATGGTGCTATGACTGCAGG
		48	2.64%	1I	T	GGTGATGGTGCTATGACTTGCAGG
		31	1.70%	6D	ATGACT	GGTGATGGTGCT------GCAGG
	#8	1005	70.63%	WT	-	GGTGATGGTGCTATGACTGCAGG
		142	9.98%	1I	T	GGTGATGGTGCTATGACTTGCAGG
		83	5.83%	1D	C	GGTGATGGTGCTATGA-TGCAGG

		80	5.62%	1I	A	GGTGATGGTGCTATGACATGCAGG
		64	4.50%	3D	TGA	GGTGATGGTGCTAT---CTGCAGG
		25	1.76%	2D	AC	GGTGATGGTGCTATG--TGCAGG
		24	1.69%	1D	T	GGTGATGGTGCTATGAC-GCAGG
	#9	1360	78.79%	WT	-	GGTGATGGTGCTATGACTGCAGG
		181	10.49%	1I	T	GGTGATGGTGCTATGACTTGCAGG
		41	2.38%	6D	ATGACT	GGTGATGGTGCT------GCAGG
		41	2.38%	1D	C	GGTGATGGTGCTATGA-TGCAGG
		32	1.85%	5D	ATGAC	GGTGATGGTGCT-----TGCAGG
		26	1.51%	2D	AC	GGTGATGGTGCTATG--TGCAGG
		23	1.33%	3D	GAC	GGTGATGGTGCTAT---TGCAGG
		22	1.27%	4D	ACTG	GGTGATGGTGCTATG----CAGG
	#11	1427	83.21%	WT	-	GGTGATGGTGCTATGACTGCAGG
		99	5.77%	1I	T	GGTGATGGTGCTATGACTTGCAGG
		87	5.07%	1I	A	GGTGATGGTGCTATGACATGCAGG
		42	2.45%	6D	ATGACT	GGTGATGGTGCT------GCAGG
		33	1.92%	1D	T	GGTGATGGTGCTATGAC-GCAGG
		27	1.57%	1D	C	GGTGATGGTGCTATGA-TGCAGG
	#12	942	80.31%	WT	-	GGTGATGGTGCTATGACTGCAGG
		76	6.48%	1I	T	GGTGATGGTGCTATGACTTGCAGG
		49	4.18%	6D	ATGACT	GGTGATGGTGCT------GCAGG
		35	2.98%	1D	T	GGTGATGGTGCTATGAC-GCAGG
		26	2.22%	1D	C	GGTGATGGTGCTATGA-TGCAGG
		25	2.13%	1I	A	GGTGATGGTGCTATGACATGCAGG
		20	1.71%	1I	G	GGTGATGGTGCTATGACGTGCAGG
	#13	952	64.72%	WT	-	GGTGATGGTGCTATGACTGCAGG
		157	10.67%	1I	T	GGTGATGGTGCTATGACTTGCAGG
		131	8.91%	1I	A	GGTGATGGTGCTATGACATGCAGG
		72	4.89%	6D	ATGACT	GGTGATGGTGCT------GCAGG
		36	2.45%	1D	C	GGTGATGGTGCTATGA-TGCAGG
		33	2.24%	4D	ACTG	GGTGATGGTGCTATG----CAGG
		26	1.77%	1D	T	GGTGATGGTGCTATGAC-GCAGG
		25	1.70%	5D	ATGAC	GGTGATGGTGCT-----TGCAGG
		20	1.36%	3D	TGA	GGTGATGGTGCTA---CTGCAGG
		19	1.29%	9D	TATGACTGC	GGTGATGGTGC---------AGG
Subgroup D	WT	1173	100.00%	WT	-	GGTGATGGTGCTATGACTGCAGG
	#1	886	81.96%	WT	-	GGTGATGGTGCTATGACTGCAGG
		97	8.97%	1I	T	GGTGATGGTGCTATGACTTGCAGG
		60	5.55%	1D	C	GGTGATGGTGCTATGA-TGCAGG
		21	1.94%	1I	C	GGTGATGGTGCTATGACCTGCAGG
		17	1.57%	1D	T	GGTGATGGTGCTATGAC-GCAGG
	#2	783	73.87%	WT	-	GGTGATGGTGCTATGACTGCAGG

	105	9.91%	1I	T	GGTGATGGTGCTATGACTTGCAGG
	75	7.08%	4D	ACTG	GGTGATGGTGCTATG----CAGG
	61	5.75%	1I	A	GGTGATGGTGCTATGACATGCAGG
	36	3.40%	6D	ATGACT	GGTGATGGTGCT------GCAGG
#4	846	70.91%	WT	-	GGTGATGGTGCTATGACTGCAGG
	132	11.06%	1I	T	GGTGATGGTGCTATGACTTGCAGG
	98	8.21%	1I	A	GGTGATGGTGCTATGACATGCAGG
	65	5.45%	6D	ATGACT	GGTGATGGTGCT------GCAGG
	32	2.68%	1D	C	GGTGATGGTGCTATGA-TGCAGG
	20	1.68%	4D	ACTG	GGTGATGGTGCTATG----CAGG
#5	1156	78.53%	WT	-	GGTGATGGTGCTATGACTGCAGG
	98	6.66%	1I	T	GGTGATGGTGCTATGACTTGCAGG
	96	6.52%	1I	A	GGTGATGGTGCTATGACATGCAGG
	55	3.74%	5D	ATGAC	GGTGATGGTGCT-----TGCAGG
	39	2.65%	3D	TGA	GGTGATGGTGCTA---CTGCAGG
	28	1.90%	9D	TATGACTGC	GGTGATGGTGC---------AGG
#6	651	88.57%	WT	-	GGTGATGGTGCTATGACTGCAGG
	35	4.76%	1I	T	GGTGATGGTGCTATGACTTGCAGG
	27	3.67%	4D	ACTG	GGTGATGGTGCTATG----CAGG
	22	2.99%	1I	A	GGTGATGGTGCTATGACATGCAGG
#7	882	64.52%	WT	-	GGTGATGGTGCTATGACTGCAGG
	228	16.68%	1I	T	GGTGATGGTGCTATGACTTGCAGG
	79	5.78%	1D	C	GGTGATGGTGCTATGA-TGCAGG
	79	5.78%	6D	ATGACT	GGTGATGGTGCT------GCAGG
	47	3.44%	1I	A	GGTGATGGTGCTATGACATGCAGG
	28	2.05%	3D	GAC	GGTGATGGTGCTAT---TGCAGG
	24	1.76%	4D	ACTG	GGTGATGGTGCTATG----CAGG
#8	862	86.11%	WT	-	GGTGATGGTGCTATGACTGCAGG
	57	5.69%	1I	T	GGTGATGGTGCTATGACTTGCAGG
	39	3.90%	1D	C	GGTGATGGTGCTATGA-TGCAGG
	26	2.60%	6D	ATGACT	GGTGATGGTGCT------GCAGG
	17	1.70%	1I	A	GGTGATGGTGCTATGACATGCAGG
#9	788	77.94%	WT	-	GGTGATGGTGCTATGACTGCAGG
	75	7.42%	1I	T	GGTGATGGTGCTATGACTTGCAGG
	47	4.65%	1D	C	GGTGATGGTGCTATGA-TGCAGG
	33	3.26%	1I	C	GGTGATGGTGCTATGACCTGCAGG
	26	2.57%	6D	ATGACT	GGTGATGGTGCT------GCAGG
	22	2.18%	4D	ACTG	GGTGATGGTGCTATG----CAGG
	20	1.98%	1I	A	GGTGATGGTGCTATGACATGCAGG
#10	613	61.48%	WT	-	GGTGATGGTGCTATGACTGCAGG

		90	9.03%	1I	T	GGTGATGGTGCTATGACTTGCAGG
		75	7.52%	1I	C	GGTGATGGTGCTATGACCTGCAGG
		56	5.62%	6D	ATGACT	GGTGATGGTGCT------GCAGG
		44	4.41%	1I	A	GGTGATGGTGCTATGACATGCAGG
		34	3.41%	1D	C	GGTGATGGTGCTATGA-TGCAGG
		26	2.61%	4D	ACTG	GGTGATGGTGCTATG----CAGG
		25	2.51%	1D	T	GGTGATGGTGCTATGAC-GCAGG
		18	1.81%	3D	GAC	GGTGATGGTGCTAT---TGCAGG
		16	1.60%	5D	ATGAC	GGTGATGGTGCT-----TGCAGG
	#11	865	74.12%	WT	-	GGTGATGGTGCTATGACTGCAGG
		81	6.94%	1I	T	GGTGATGGTGCTATGACTTGCAGG
		65	5.57%	3D	GAC	GGTGATGGTGCTAT---TGCAGG
		47	4.03%	2D	AC	GGTGATGGTGCTATG--TGCAGG
		36	3.08%	1I	C	GGTGATGGTGCTATGACCTGCAGG
		31	2.66%	1D	T	GGTGATGGTGCTATGAC-GCAGG
		23	1.97%	1D	C	GGTGATGGTGCTATGA-TGCAGG
		19	1.63%	1I	A	GGTGATGGTGCTATGACATGCAGG
	#12	783	75.43%	WT	-	GGTGATGGTGCTATGACTGCAGG
		85	8.19%	1I	T	GGTGATGGTGCTATGACTTGCAGG
		31	2.99%	6D	ATGACT	GGTGATGGTGCT------GCAGG
		31	2.99%	1D	C	GGTGATGGTGCTATGA-TGCAGG
		26	2.50%	1D	T	GGTGATGGTGCTATGAC-GCAGG
		25	2.41%	4D	ACTG	GGTGATGGTGCTATG----CAGG
		22	2.12%	1I	C	GGTGATGGTGCTATGACCTGCAGG
		18	1.73%	1I	G	GGTGATGGTGCTATGACGTGCAGG
		17	1.64%	1I	A	GGTGATGGTGCTATGACATGCAGG

WT is the control; SNP indicates base substitution; D indicates base deletion; I indicates base insertion

4. High-throughput sequencing results for CLCrV-*GhCLA1*-sgRNA.

Genome	Plant number	Number of sample reads	Mutation ratio	Mutation type	Mutations in the sequence	Mutation site
Subgroup A	WT	1623	100.00%	WT	-	GGTGATGGTGCTATGACTGCAGG
	#1	1231	78.26%	WT	-	GGTGATGGTGCTATGACTGCAGG
		127	8.07%	1D	T	GGTGATGGTGCTATGAC-GCAGG
		87	5.53%	1D	C	GGTGATGGTGCTATGA-TGCAGG
		67	4.26%	6D	ATGACT	GGTGATGGTGCT------GCAGG
		32	2.03%	2D	AC	GGTGATGGTGCTATG--TGCAGG
		29	1.84%	1I	A	GGTGATGGTGCTATGACATGCAGG
	#2	1141	85.28%	WT	-	GGTGATGGTGCTATGACTGCAGG
		75	5.61%	2D	AC	GGTGATGGTGCTATG--TGCAGG
		49	3.66%	2D	CT	GGTGATGGTGCTATGA--GCAGG

	38	2.84%	1D	C	GGTGATGGTGCTATGA-TGCAGG
	35	2.62%	6D	ATGACT	GGTGATGGTGCT------GCAGG
#4	1367	90.35%	WT	-	GGTGATGGTGCTATGACTGCAGG
	72	4.76%	3D	TGA	GGTGATGGTGCTA---CTGCAGG
	53	3.50%	1D	T	GGTGATGGTGCTATGAC-GCAGG
	21	1.39%	2D	CT	GGTGATGGTGCTATGA--GCAGG
#5	990	72.26%	WT	-	GGTGATGGTGCTATGACTGCAGG
	88	6.42%	5D	ATGAC	GGTGATGGTGCT-----TGCAGG
	86	6.28%	1D	T	GGTGATGGTGCTATGAC-GCAGG
	62	4.53%	6D	ATGACT	GGTGATGGTGCT------GCAGG
	47	3.43%	1I	T	GGTGATGGTGCTATGACTTGCAGG
	31	2.26%	4D	ACTG	GGTGATGGTGCTATG----CAGG
	27	1.97%	1I	A	GGTGATGGTGCTATGACATGCAGG
	22	1.61%	1D	C	GGTGATGGTGCTATGA-TGCAGG
	17	1.24%	2D	AC	GGTGATGGTGCTATG--TGCAGG
#6	955	71.59%	WT	-	GGTGATGGTGCTATGAC TGCAGG
	119	8.92%	1D	C	GGTGATGGTGCTATGA-TGCAGG
	77	5.77%	2D	AC	GGTGATGGTGCTATG--TGCAGG
	42	3.15%	2D	CT	GGTGATGGTGCTATGA--GCAGG
	34	2.55%	1I	T	GGTGATGGTGCTATGACTTGCAGG
	27	2.02%	1D	T	GGTGATGGTGCTATGAC-GCAGG
	24	1.80%	6D	ATGACT	GGTGATGGTGCT------GCAGG
	20	1.50%	4D	ACTG	GGTGATGGTGCTATG----CAGG
	19	1.42%	1I	A	GGTGATGGTGCTATGACATGCAGG
	17	1.27%	3D	GAC	GGTGATGGTGCTAT---TGCAGG
#7	977	78.66%	WT	-	GGTGATGGTGCTATGACTGCAGG
	202	16.26%	1I	T	GGTGATGGTGCTATGACTTGCAGG
	42	3.38%	1D	C	GGTGATGGTGCTATGA-TGCAGG
	21	1.69%	1D	T	GGTGATGGTGCTATGAC-GCAGG
#8	1441	84.47%	WT	-	GGTGATGGTGCTATGACTGCAGG
	65	3.81%	2D	AC	GGTGATGGTGCTATG--TGCAGG
	54	3.17%	9D	TATGACTGC	GGTGATGGTGC---------AGG
	46	2.70%	4D	ACTG	GGTGATGGTGCTATG----CAGG
	44	2.58%	1I	T	GGTGATGGTGCTATGACTTGCAGG
	31	1.82%	1D	T	GGTGATGGTGCTATGAC-GCAGG
	25	1.47%	3D	GAC	GGTGATGGTGCTAT---TGCAGG
#9	1268	91.35%	WT	-	GGTGATGGTGCTATGACTGCAGG
	65	4.68%	1D	T	GGTGATGGTGCTATGAC-GCAGG
	33	2.38%	3D	GAC	GGTGATGGTGCTAT---TGCAGG
	22	1.59%	4D	ACTG	GGTGATGGTGCTATG----CAGG
#10	1104	82.02%	WT	-	GGTGATGGTGCTATGACTGCAGG
	50	3.71%	2D	AC	GGTGATGGTGCTATG--TGCAGG
	39	2.90%	3D	TGA	GGTGATGGTGCTA---CTGCAGG

		38	2.82%	6D	ATGACT	GGTGATGGTGCT------GCAGG
		33	2.45%	1D	T	GGTGATGGTGCTATGAC-GCAGG
		26	1.93%	1I	T	GGTGATGGTGCTATGACTTGCAGG
		20	1.49%	1I	A	GGTGATGGTGCTATGACATGCAGG
		19	1.41%	3D	GAC	GGTGATGGTGCTAT---TGCAGG
		17	1.26%	4D	ACTG	GGTGATGGTGCTATG----CAGG
	#11	1035	75.88%	WT	-	GGTGATGGTGCTATGACTGCAGG
		97	7.11%	1I	T	GGTGATGGTGCTATGACTTGCAGG
		71	5.21%	4D	ACTG	GGTGATGGTGCTATG----CAGG
		56	4.11%	1D	T	GGTGATGGTGCTATGAC-GCAGG
		45	3.30%	5D	ATGAC	GGTGATGGTGCT-----TGCAGG
		37	2.71%	2D	AC	GGTGATGGTGCTATG--TGCAGG
		23	1.69%	1I	C	GGTGATGGTGCTATGACCTGCAGG
Subgroup D	WT	1226	100.00%	WT	-	GGTGATGGTGCTATGACTGCAGG
	#1	975	77.32%	WT	-	GGTGATGGTGCTATGACTGCAGG
		87	6.90%	1D	T	GGTGATGGTGCTATGAC-GCAGG
		56	4.44%	1D	C	GGTGATGGTGCTATGA-TGCAGG
		43	3.41%	4D	ACTG	GGTGATGGTGCTATG----CAGG
		35	2.78%	5D	ATGAC	GGTGATGGTGCT-----TGCAGG
		27	2.14%	6D	ATGACT	GGTGATGGTGCT------GCAGG
		20	1.59%	3D	TGA	GGTGATGGTGCTA---CTGCAGG
		18	1.43%	2D	AC	GGTGATGGTGCTATG--TGCAGG
	#2	678	70.85%	WT	-	GGTGATGGTGCTATGACTGCAGG
		131	13.69%	1I	T	GGTGATGGTGCTATGACTTGCAGG
		75	7.84%	4D	ACTG	GGTGATGGTGCTATG----CAGG
		34	3.55%	6D	ATGACT	GGTGATGGTGCT------GCAGG
		22	2.30%	1D	C	GGTGATGGTGCTATGA-TGCAGG
		17	1.78%	1I	A	GGTGATGGTGCTATGACATGCAGG
	#3	854	65.04%	WT	-	GGTGATGGTGCTATGACTGCAGG
		255	19.42%	2D	CT	GGTGATGGTGCTATGA--GCAGG
		101	7.69%	3D	TGA	GGTGATGGTGCTA---CTGCAGG
		44	3.35%	2D	AC	GGTGATGGTGCTATG--TGCAGG
		23	1.75%	1D	T	GGTGATGGTGCTATGAC-GCAGG
		20	1.52%	1D	C	GGTGATGGTGCTATGA-TGCAGG
		16	1.22%	4D	ACTG	GGTGATGGTGCTATG----CAGG
	#4	763	63.27%	WT	-	GGTGATGGTGCTATGACTGCAGG
		201	16.67%	3D	TGA	GGTGATGGTGCTA---CTGCAGG
		82	6.80%	9D	TATGACTGC	GGTGATGGTGC---------AGG
		76	6.30%	1I	T	GGTGATGGTGCTATGACTTGCAGG
		45	3.73%	3D	GAC	GGTGATGGTGCTAT---TGCAGG
		20	1.66%	1D	T	GGTGATGGTGCTATGAC-GCAGG

	19	1.58%	5D	ATGAC	GGTGATGGTGCT-----TGCAGG
#5	1094	96.73%	WT	-	GGTGATGGTGCTATGACTGCAGG
	37	3.27%	2D	AC	GGTGATGGTGCTATG--TGCAGG
#6	648	78.26%	WT	-	GGTGATGGTGCTATGACTGCAGG
	41	4.95%	1D	C	GGTGATGGTGCTATGA-TGCAGG
	33	3.99%	1I	T	GGTGATGGTGCTATGACTTGCAGG
	32	3.86%	1I	A	GGTGATGGTGCTATGACATGCAGG
	30	3.62%	3D	GAC	GGTGATGGTGCTAT---TGCAGG
	23	2.78%	4D	ACTG	GGTGATGGTGCTATG----CAGG
	21	2.54%	2D	AC	GGTGATGGTGCTATG--TGCAGG
#7	752	69.69%	WT	-	GGTGATGGTGCTATGACTGCAGG
	286	26.51%	1I	T	GGTGATGGTGCTATGACTTGCAGG
	22	2.04%	1D	C	GGTGATGGTGCTATGA-TGCAGG
	19	1.76%	4D	ACTG	GGTGATGGTGCTATG----CAGG
#8	851	65.41%	WT	-	GGTGATGGTGCTATGACTGCAGG
	307	23.60%	1I	T	GGTGATGGTGCTATGACTTGCAGG
	46	3.54%	4D	ACTG	GGTGATGGTGCTATG----CAGG
	29	2.23%	6D	ATGACT	GGTGATGGTGCT------GCAGG
	27	2.08%	1D	C	GGTGATGGTGCTATGA-TGCAGG
	22	1.69%	9D	TATGACTGC	GGTGATGGTGC---------AGG
	19	1.46%	1I	A	GGTGATGGTGCTATGACATGCAGG
#9	846	69.06%	WT	-	GGTGATGGTGCTATGACTGCAGG
	93	7.59%	1D	C	GGTGATGGTGCTATGA-TGCAGG
	82	6.69%	1D	T	GGTGATGGTGCTATGAC-GCAGG
	37	3.02%	2D	AC	GGTGATGGTGCTATG--TGCAGG
	33	2.69%	2D	CT	GGTGATGGTGCTATGA--GCAGG
	31	2.53%	6D	ATGACT	GGTGATGGTGCT------GCAGG
	29	2.37%	4D	ACTG	GGTGATGGTGCTATG----CAGG
	26	2.12%	5D	ATGAC	GGTGATGGTGCT-----TGCAGG
	26	2.12%	1I	T	GGTGATGGTGCTATGACTTGCAGG
	22	1.80%	3D	GAC	GGTGATGGTGCTAT---TGCAGG
#10	1231	77.71%	WT	-	GGTGATGGTGCTATGACTGCAGG
	87	5.49%	1I	T	GGTGATGGTGCTATGACTTGCAGG
	85	5.37%	3D	TGA	GGTGATGGTGCTA---CTGCAGG
	67	4.23%	1I	A	GGTGATGGTGCTATGACATGCAGG
	54	3.41%	1D	T	GGTGATGGTGCTATGAC-GCAGG
	36	2.27%	2D	AC	GGTGATGGTGCTATG--TGCAGG
	24	1.52%	4D	ACTG	GGTGATGGTGCTATG----CAGG
#11	881	75.75%	WT	-	GGTGATGGTGCTATGACTGCAGG
	87	7.48%	3D	TGA	GGTGATGGTGCTA---CTGCAGG

	83	7.14%	2D	AC	GGTGATGGTGCTATG--TGCAGG
	67	5.76%	1D	T	GGTGATGGTGCTATGAC-GCAGG
	26	2.24%	9D	TATGACTGC	GGTGATGGTGC---------AGG
	19	1.63%	3D	GAC	GGTGATGGTGCTAT---TGCAGG
#12	797	55.66%	WT	-	GGTGATGGTGCTATGACTGCAGG
	271	18.92%	1I	T	GGTGATGGTGCTATGACTTGCAGG
	134	9.36%	3D	TGA	GGTGATGGTGCTA---CTGCAGG
	95	6.63%	1I	A	GGTGATGGTGCTATGACATGCAGG
	77	5.38%	1D	T	GGTGATGGTGCTATGAC-GCAGG
	36	2.51%	2D	AC	GGTGATGGTGCTATG--TGCAGG
	22	1.54%	4D	ACTG	GGTGATGGTGCTATG----CAGG

WT is the control; SNP indicates base substitution; D indicates base deletion; I indicates base insertion
